# Regulation of human neutrophil IL-1β secretion induced by *Escherichia coli* O157:H7 responsible for hemolytic uremic syndrome

**DOI:** 10.1371/journal.ppat.1011877

**Published:** 2023-12-21

**Authors:** Florencia Sabbione, Irene Angelica Keitelman, Carolina Maiumi Shiromizu, Alexia Vereertbrugghen, Douglas Vera Aguilar, Paolo Nahuel Rubatto Birri, Manuela Pizzano, María Victoria Ramos, Federico Fuentes, Lucas Saposnik, Agostina Cernutto, Juliana Cassataro, Carolina Cristina Jancic, Jeremías Gaston Galletti, Marina Sandra Palermo, Analía Silvina Trevani

**Affiliations:** 1 Laboratorio de inmunidad innata, Instituto de Medicina Experimental (IMEX)—CONICET, Academia Nacional de Medicina, Buenos Aires, Argentina; 2 Laboratorio de patogénesis e inmunología de procesos infecciosos. Instituto de Medicina Experimental (IMEX)—CONICET, Academia Nacional de Medicina, Buenos Aires, Argentina; 3 Laboratorio de microscopía, Instituto de Medicina Experimental (IMEX)—CONICET, Academia Nacional de Medicina, Buenos Aires, Argentina; 4 Instituto de Investigaciones Biotecnológicas, Universidad Nacional de San Martín (UNSAM)–Consejo Nacional de Investigaciones Científicas y Técnicas (CONICET), Universidad Nacional de San Martín. San Martín, Buenos Aires, Argentina; 5 Escuela de Bio y Nanotecnologías (EByN), Universidad Nacional de San Martín. San Martín, Buenos Aires, Argentina; 6 Departamento de Microbiología, Parasitología e Inmunología, Facultad de Medicina, Universidad de Buenos Aires, Buenos Aires, Argentina; University of California Davis School of Medicine, UNITED STATES

## Abstract

Shiga-toxin producing *Escherichia coli* (STEC) infections can cause from bloody diarrhea to Hemolytic Uremic Syndrome. The STEC intestinal infection triggers an inflammatory response that can facilitate the development of a systemic disease. We report here that neutrophils might contribute to this inflammatory response by secreting Interleukin 1 beta (IL-1β). STEC stimulated neutrophils to release elevated levels of IL-1β through a mechanism that involved the activation of caspase-1 driven by the NLRP3-inflammasome and neutrophil serine proteases (NSPs). Noteworthy, IL-1β secretion was higher at lower multiplicities of infection. This secretory profile modulated by the bacteria:neutrophil ratio, was the consequence of a regulatory mechanism that reduced IL-1β secretion the higher were the levels of activation of both caspase-1 and NSPs, and the production of NADPH oxidase-dependent reactive oxygen species. Finally, we also found that inhibition of NSPs significantly reduced STEC-triggered IL-1β secretion without modulating the ability of neutrophils to kill the bacteria, suggesting NSPs might represent pharmacological targets to be evaluated to limit the STEC-induced intestinal inflammation.

## Author summary

The enteric pathogen Shiga toxin (Stx)-producing *Escherichia coli* (STEC) is a food- and water-borne bacteria that upon ingestion colonizes the intestine where it releases Shiga-toxins (Stx) and other virulence factors. STEC not only can cause self-limited gastrointestinal infections and bloody diarrhea but also a severe and potentially deathly systemic condition, known as Hemolytic Uremic Syndrome (HUS). There are no vaccines available to prevent STEC infections or specific treatments. The most frequent clinical STEC isolate in HUS is *E*. *coli* O157:H7. Severe inflammation and damage to the mucosal membrane are common symptoms of gastroenteritis induced by STEC. Interleukin-1β (IL-1β) is a highly pro-inflammatory mediator. Neutrophils are professional phagocytes that are extensively recruited to the intestinal mucosa upon STEC infection. Here we determined that human neutrophils secrete IL-1β in response to STEC and identified a molecular mechanism that limits the secretion of this mediator as the dose of bacteria that interacts with the neutrophils increases. Our study provides new insights into the role of neutrophils in the inflammatory response against STEC infections and opens a new road to investigate therapeutic strategies to avoid the progression to HUS.

## Introduction

The enteric pathogen Shiga toxin (Stx)-producing *Escherichia coli* (STEC) remains a major public health concern because it not only causes self-limited gastrointestinal infections and bloody diarrhea, but also a severe systemic condition known as Hemolytic Uremic Syndrome (HUS) in 5–15% of cases (and even in 20–30% during outbreaks) [1]. HUS is characterized by the triad of non-immune microangiopathic hemolytic anemia, thrombocytopenia, and acute kidney injury. In Latin America, enterohemorragic *E*. *coli* infections remain endemic and contribute to the burden of the acute diarrheal syndrome. Particularly in Argentina, STEC infections and HUS also show an endemic pattern with high morbimortality rates, especially in children younger than 5 years [2]. Due to the absence of vaccines and interventional therapies, HUS represents the most common cause of acute renal failure in early childhood [3]. Even though extra-renal manifestations in HUS are less frequent, they dictate the prognosis in children [4]. Cerebral compromise is also observed in HUS cases being more common in adults than in children. Although unusual, cardiac involvement is a cause of severe disease and death [4].

STEC colonizes cattle and transmission is fecal-to-oral via undercooked contaminated meat, raw milk products, drinking water, direct contact with animals, and person-to-person spread [1,2]. The O157:H7 STEC is the predominant serotype that causes HUS worldwide. However, sporadic epidemics also occur, like the one in Germany in 2011 caused by the O104:H4 serotype, associated with frequent neurological complications and high mortality [1,5].

STEC are non-invasive pathogens that colonize the intestinal mucosa releasing Stx, their major virulence factor, which is translocated to the bloodstream reaching its target organs. STEC can produce two types of Stx, type 1 (Stx1) and type 2 (Stx2); being Stx2 the associated with more severe toxicity [1,5]. Evidence suggests that Stx is translocated across the epithelial barrier by transcellular transcytosis via receptor-independent macropinocytosis during the early stages of infection [6], while the paracellular pathway is suspected to be the mechanism during the acute inflammation of the colonic mucosa [5]. In fact, STEC infection induce extensive neutrophil infiltration in the lamina propria as well as an increased frequency of fecal leukocytes which suggest its transmigration across the epithelium into the intestinal lumen [7,8]. In vitro studies showed that Stx apical-to-basolateral movement through the epithelium increases during the process of neutrophil migration from the basolateral-to the apical surface, suggesting that neutrophil epithelial transmigration might increase the risk of HUS [9]. In line with this possibility, neutrophilia is usually found in patients with HUS, and a high peripheral blood neutrophil count at presentation is the poor-prognosis factor most consistently reported in this disease [10]. Furthermore, the STEC-induced gastroenteritis is accompanied by marked inflammation and mucosal damage in the caecum and ascending colon [7], and it has been proposed that Stx leaks through damaged epithelium at later stages of infection [7].

Neutrophils have been considered for many years only as microbicidal cells. However, evidence from the last two decades indicates their functions go well beyond just pathogen killing [11]. These cells also produce and release a variety of cytokines [12]. Previously, we determined that in response to lipopolysaccharide (LPS) plus ATP, neutrophils secrete the potent pro-inflammatory cytokine Interleukin-1β (IL-1β) through an unconventional autophagy secretory mechanism [13].

Interleukin-1β is synthesized as a leaderless inactive precursor (pro-IL-1β) in the cytosol, where it undergoes enzymatic cleavage to achieve an active isoform [14]. Usually, pro-IL-1β processing is mediated by caspase-1. This enzyme is activated within macromolecular platforms called inflammasomes. These macromolecular complexes are composed of a sensor molecule (a pattern recognition receptor; PRR) that detects pathogen- or danger-associated molecular patterns (PAMPs or DAMPs), and/or perturbations in cellular homeostasis, and usually, the adaptor protein ASC (apoptosis-associated speck-like protein containing the caspase activation and recruitment domain [CARD]), that bridges the interaction between the stimulus sensing-PRR and caspase-1 [15]. One of such sensors is NLRP3, which triggers its oligomerization and ASC recruitment and polymerization upon activation, originating an ASC-speck structure to which caspase-1 attaches and dimerizes. This event leads to the activation by auto-processing of caspase-1, which remains bound to this hub and is responsible for processing of both pro-IL-1β and the latent pore-forming protein Gasdermin D (GSDMD). Then, caspase-1 undergoes a self-cleavage event that produces a species that is released from the inflammasome and ceases its protease activity [16].

Although caspase-1 is responsible for pro-IL-1β processing in most myeloid cells, our previous data in human neutrophils indicated that in response to LPS+ATP this enzyme is required for IL-1β release and has the capacity to process its precursor, but the neutrophil serine proteases (NSPs) exert a major role in this task [17]. Thus, in this study, with the aim to elucidate if neutrophils might contribute to the STEC-induced proinflammatory response by secreting IL-1β, we evaluated their ability to secrete this cytokine in response to the highly pathogenic O157:H7 STEC strain [18] and investigated the mechanisms involved in this response.

## Results

### Viable STEC induces human neutrophil IL-1β secretion independently of its capacity to produce Shiga toxin

**Fig 1:**
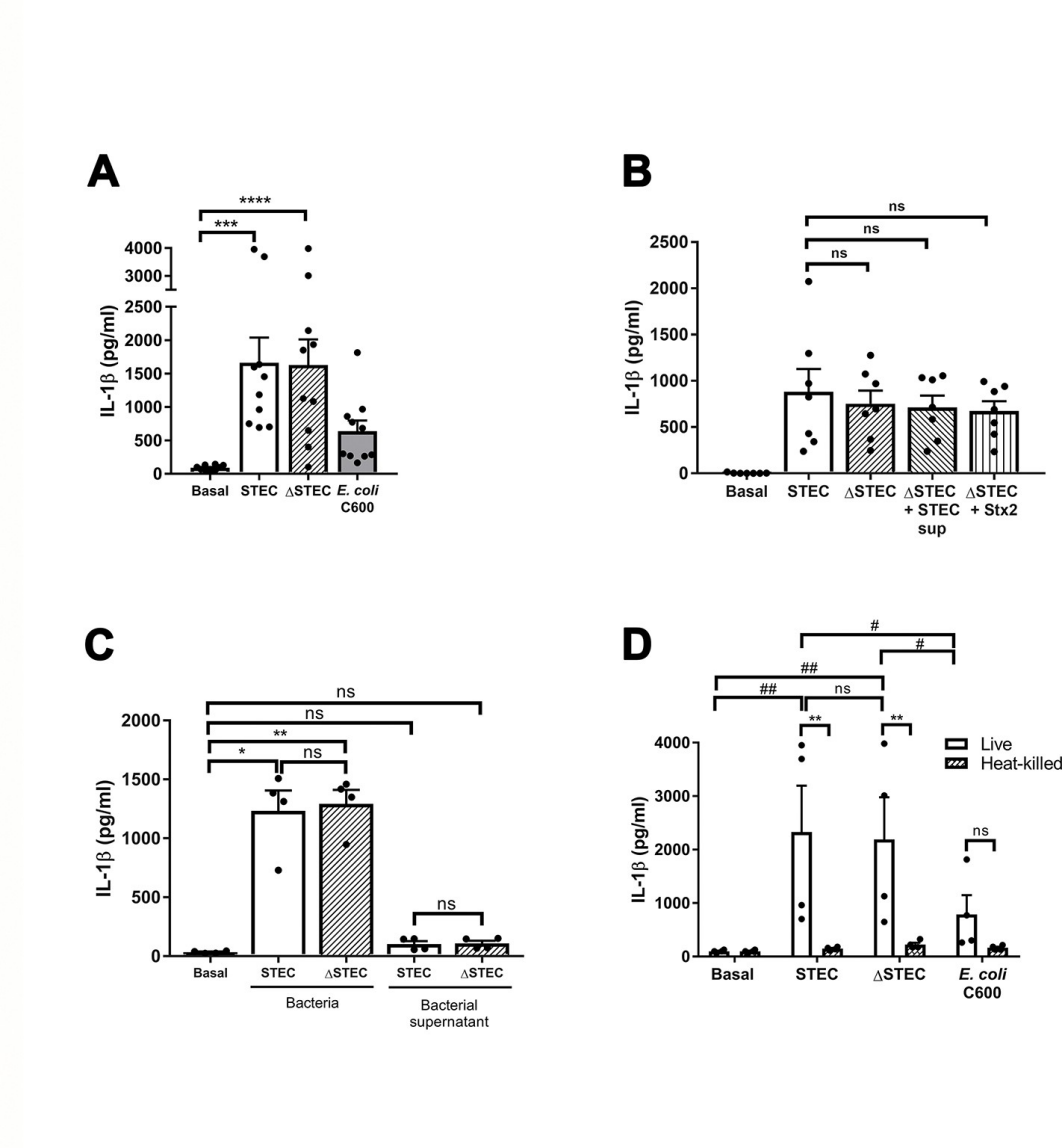
Viable STEC stimulates human neutrophil IL-1β secretion independently of its capacity to produce Shiga toxin. Neutrophils were cultured for 3.5 h at 37°C and 5% CO_2_ (**A**) without (basal) or with Stx2-producing *E*. *coli* O157:H7 (125/99; STEC), *E*. *coli* O157:H7ΔStx2 devoid of Stx producing capacity (ΔSTEC), or *E*. *coli* C600 (a non-pathogenic strain) at a MOI of 0.5; (**B**) without (basal) or with STEC, ΔSTEC, ΔSTEC whose supernatant was previously replaced by the medium from a STEC culture (ΔSTEC+STEC sup), or ΔSTEC supplemented with purified Stx2 (0.1 μg/ml; ΔSTEC+Stx2) at MOI 0.5; and the concentrations of IL-1β (**A** and **B**) in culture supernatants were determined. (**C**) Neutrophils were cultured for 3.5 h at 37°C and 5% CO_2_ without (basal) or with STEC or ΔSTEC at MOI 0.5, or with the bacterial supernatants alone; and (**D**) without (basal) or with viable or heat-killed STEC, ΔSTEC, or *E*. *coli* C600, and the concentrations of IL-1β in culture supernatants were determined by ELISA. Graphs depict the mean ± SEM of experiments performed in triplicate; each dot represents the triplicate´s mean for each individual donor sample. Statistical significance between samples was assessed by One-way ANOVA followed by Sidak´s multiple comparisons test for data with normal distribution and Friedman test followed by Dunn’s multiple comparisons test for data with non-normal distribution, except for (**D**) in which data were analyzed by Two-way ANOVA. *or # p<0.05; ** or ## p<0.01; ***p<0.001; ****p<0.0001; ns: non-significant.

Considering STEC can establish infections at very low doses (1–100 colony forming units; CFU) [19], we first challenged highly pure human neutrophils with the O157:H7 STEC strain (1:2 bacteria: neutrophil ratio; multiplicity of infection -MOI- 0.5) and evaluated IL-1β concentrations in culture supernatants. As shown in Fig 1A, STEC induced high levels of IL-1β secretion. This property was not associated with the ability of the bacteria to produce Stx, as we found similar IL-1β levels when neutrophils were challenged with a strain devoid of the capacity to produce this toxin -ΔSTEC- (Fig 1A). This was confirmed by assays in which the culture supernatant of ΔSTEC was replaced by supernatants of the same amount of STEC bacteria before challenging neutrophils (Fig 1B). In these assays neutrophils released similar IL-1β levels to those secreted in response to STEC, as did when challenged with a ΔSTEC culture supplemented with 0.1 μg/ml of exogenous Stx2 (Fig 1B). Moreover, bacterial supernatant from STEC or the ΔSTEC isogenic mutant did not induce IL-1β secretion (Fig 1C). Altogether, these results exclude that Stx is responsible for stimulating neutrophil IL-1β secretion induced by STEC. However, other bacterial virulence factors appear to contribute to the stimulation of neutrophil IL-1β secretion, because challenge with a non-pathogenic *E*. *coli* strain (C600) resulted in markedly lower levels of IL-1β secretion, which did not reach statistical significance (Fig 1A). Of note, the differences between strains to stimulate neutrophil IL-1β secretion were not due to a distinct capacity to modulate neutrophil viability, as we detected similar LDH levels in the supernatants at the end of the coculture under all the conditions (S1A and S1B Fig).

Additional assays showed a requirement of bacterial viability to stimulate neutrophil IL-1β secretion as only live- but not heat-killed-bacteria significantly increased IL-1β levels in culture supernatants (Fig 1D).

### Neutrophil IL-1β secretion induced by STEC involves the NLRP3 inflammasome and caspase-1 activation

**Fig 2:**
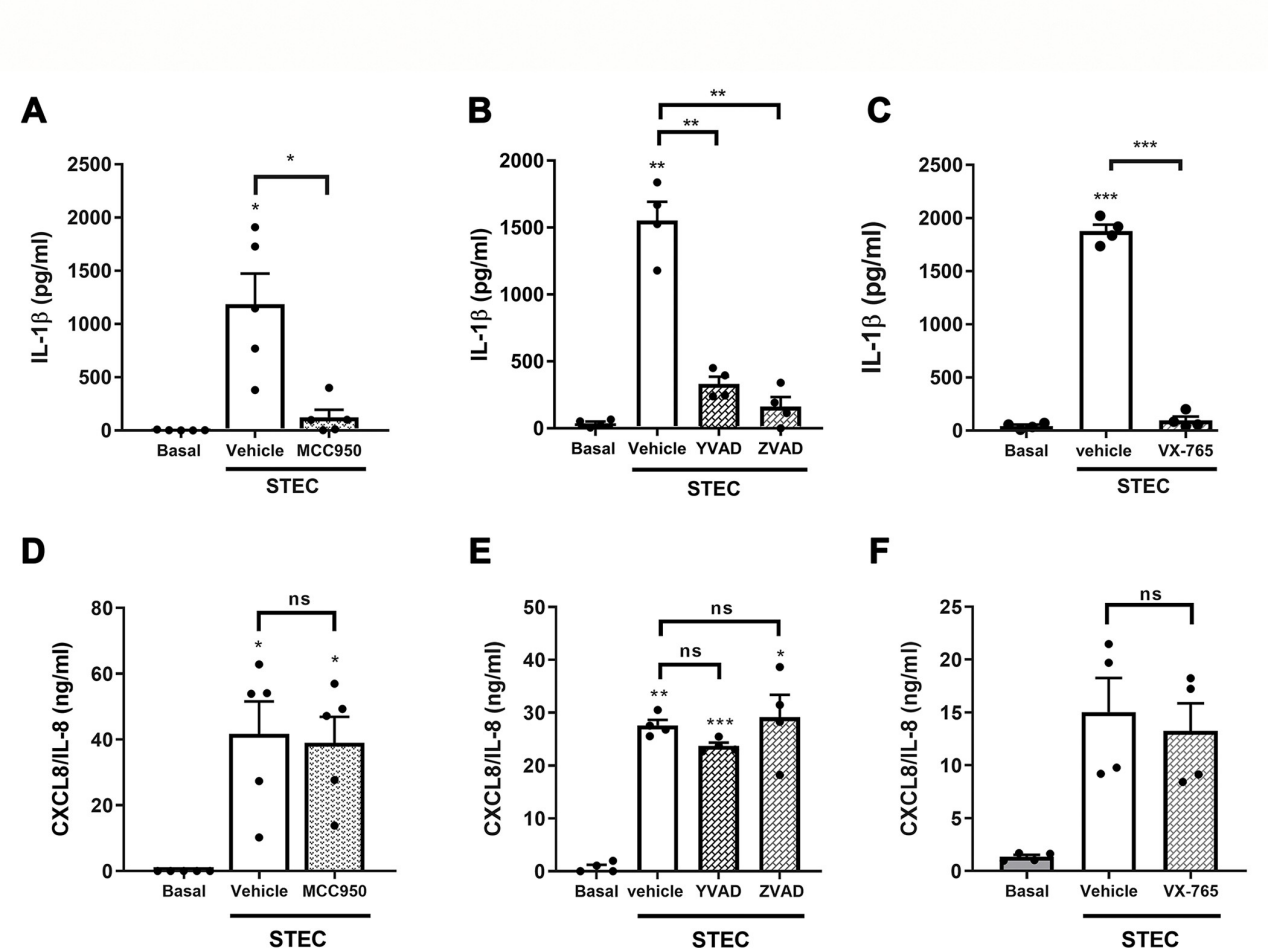
Human neutrophil IL-1β secretion induced by STEC depends on NLRP3 inflammasome activation and caspase-1 activity. Neutrophils were pretreated or not with (**A and D**) the NLRP3 inhibitor (MCC950, 10 μM); (**B and E**) the caspase-1 inhibitor Ac-YVAD-CMK (YVAD; 50 μM) or the PAN-caspase inhibitor Z-VAD-FMK (ZVAD, 50μM); or (**C and F**) the caspase-1/4 inhibitor VX-765 (50 μM), for 30 min and then challenged with STEC (MOI 0.5) for 3.5 h at 37°C and 5% CO_2_. IL-1β (**A-C**) and CXCL8/IL-8 concentrations (**D-F**) in culture supernatants were determined by ELISA. Graphs depict the mean ± SEM of experiments performed in triplicate; each dot represents the triplicate´s mean for each individual donor sample. Statistical significance between samples was assessed by One-way ANOVA followed by Sidak´s multiple comparisons test for data with normal distribution and Friedman test followed by Dunn’s multiple comparisons test for data with non-normal distribution. *p<0.05; **p<0.01; ***p<0.001; ns: non-significant.

To get insight into the mechanisms underlying IL-1β secretion in response to STEC, we investigated the effects of pre-treating neutrophils with inhibitors targeting two different steps of the inflammasome pathway. These included the potent NLRP3 inflammasome inhibitor MCC950, the caspase-1 inhibitor Ac-YVAD-CMK, the caspase-1/4 inhibitor VX-765, and the PAN-caspase inhibitor Z-VAD-FMK. As shown in Fig 2, all the inhibitors marked- and significantly reduced IL-1β levels in culture supernatants (Fig 2A–2C). However, none of the inhibitors affected the secretion of CXCL8/IL-8, another cytokine produced in response to STEC, that does not require inflammasome activation for processing (Fig 2D–2F). Moreover, the effect of the inhibitors was not due to a toxic activity as reduced and similar LDH levels were detected in culture supernatants of cocultures independently of the treatment (S2A–S2C Fig). Altogether, these findings indicate that neutrophil IL-1β secretion induced by STEC involves the NLRP3 inflammasome activation and caspase-1 activity.

### Neutrophil IL-1β secretion upon STEC challenge decreases as the multiplicity of infection (MOI) increases and this profile is independent on Shiga toxin production by the bacterium

**Fig 3:**
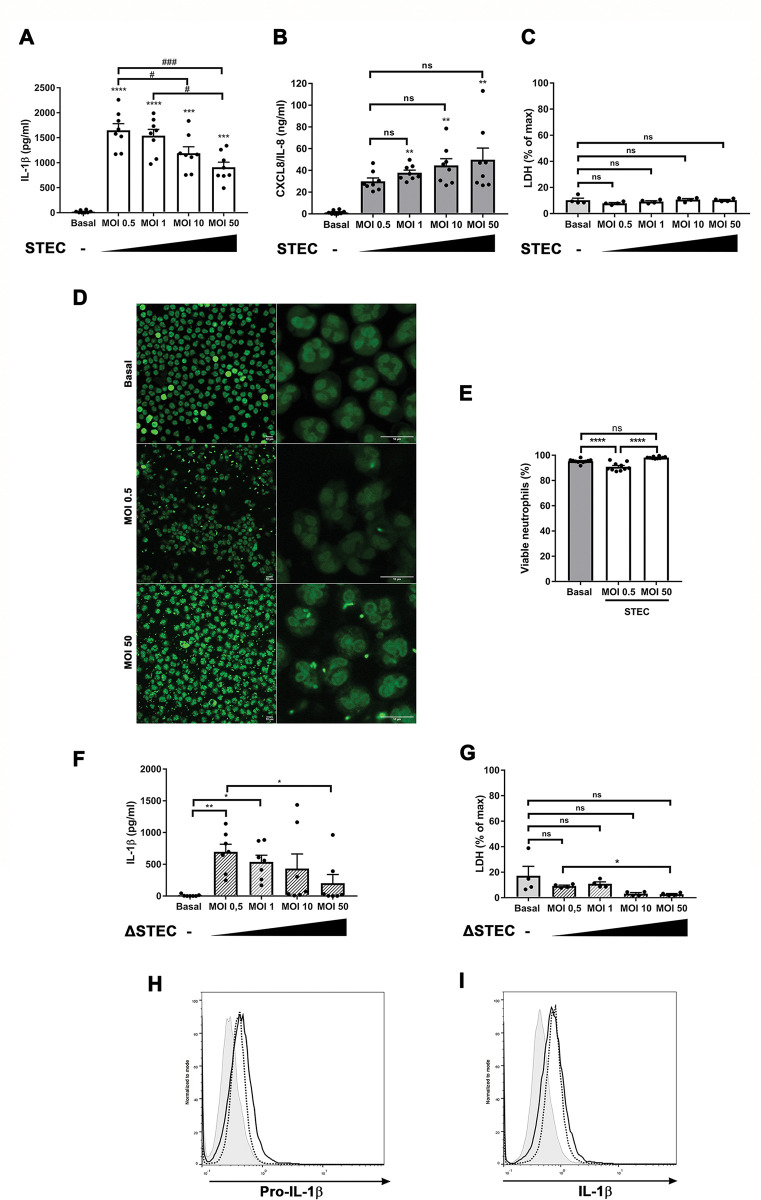
The human neutrophil IL-1β secretory response, in contrast to the CXCL8/IL-8 secretory response decreases as MOI increases. Neutrophils were cultured for 3.5 h at 37°C and 5% CO_2_ without (basal) or with STEC (**A-D**) or ΔSTEC (**F and G**) at the indicated MOI (0.5–50). Then, IL-1β (**A** and **F**) and CXCL8/IL-8 (**B**) concentrations in culture supernatants were determined by ELISA, or LDH activity in culture supernatants (**C** and **G**) were examined by an LDH Cytotoxicity Assay Kit. Graphs depict the mean ± SEM of experiments performed in triplicate; each dot represents the triplicate´s mean for each individual donor sample. Statistical significance between samples was assessed by One-way ANOVA followed by Sidak´s multiple comparisons test for data with normal distribution and Friedman test followed by Dunn’s multiple comparisons test for data with non-normal distribution. *and # p<0.05; **p<0.01; *** and ###p<0.001; ****p<0.0001; ns: non-significant. (**A** and **B**) asterisks represent comparisons vs basal. (**D**) Confocal microscopy images of neutrophils cultured for 3.5 h at 37°C and 5% CO_2_ without (basal) or with STEC at the indicated MOI, then fixed and stained with propidium iodide. Cells were pseudo-colored in green. Bars represent 10 μm. (**E**) The percentage of cells with lobulated nuclei, considered as a parameter for viable cells, was quantitated from 10 microscopic fields for condition per donor from images captured by confocal microscopy as shown in **D**. Each dot represents the mean number of cells of one microscopic field. One representative donor of two analyzed is shown. (**H and I**) Representative histograms of Pro-IL-1β (**H**) and IL-1β (I) expression in neutrophils challenged with STEC at a MOI of 0.5 (dotted black line) or MOI of 50 (solid black line) for 2 h at 37°C and 5% CO_2_ evaluated by immunostaining and flow cytometry. Grey tinted histogram: isotype control. Histograms are representative of experiments with 4 donors.

Speculating that once in the gut, the bacteria would replicate increasing the number of organisms that the recruited neutrophils would face, we then evaluated the IL-1β response upon exposure of neutrophils to STEC at different MOI. Surprisingly, we found that IL-1β secretion significantly decreased as MOI increased from 0.5 to 50 (Fig 3A). This behavior was particular to IL-1β because CXCL8/IL-8 did not show the same pattern (Fig 3B). Of note, the reduction in IL-1β secretion observed at higher MOI was not due to differences in neutrophil viability as similar LDH levels were detected in culture supernatants at the end of the coculture (Fig 3C). These findings were confirmed by confocal microscopy of live cells cultured in the presence of SYTOX green, a DNA stain that only enters cells with compromised membranes (S3 Fig). Moreover, confocal microscopy examination of neutrophil incubated with STEC for 3.5 h and stained with propidium iodide after fixation, showed that most neutrophils retained their lobulated nuclei, indicating they had not undergone apoptosis either (Figs 3D, 3E, and S4). Additionally, IL-1β secretion at MOI 0.5 and 50 did not differ at an earlier time point (2 h) (S5 Fig). This indicates that the decrease in IL-1β extracellular levels at higher MOI in Fig 3A was not due to an accelerated kinetic of secretion followed by degradation of the cytokine.

To determine if the reduction in IL-1β secretion observed at high MOI was due to an increase in Stx concentration in contact with neutrophils, we evaluated the IL-1β neutrophil response induced by the *E*. *coli* strain unable to produce Stx (ΔSTEC). As expected according to data shown in Fig 1, a reduction in IL-1β secretion was also detected as MOI increased from 0.5 to 50 (Fig 3F), and this effect was not associated with lytic cell death (Fig 3G).

We further evaluated if the reduction in IL-1β secretion observed at high MOI was the result of an inhibition of the pro-IL-1β synthesis or its processing, by determining their levels by intracellular immunostaining and flow cytometry at an early time-point (2 h). As shown in Fig 3H and 3I, we did not detect lower levels of both isoforms at MOI 50 regarding those observed at MOI 0.5. Altogether, these results suggest that higher MOI of STEC stimulate lower levels of IL-1β secretion by neutrophils, an effect that is neither associated with the capacity of high bacterial counts to produce more Stx nor with differences in pro-IL-1β synthesis.

### IL-1β secretion induced by STEC requires neutrophil serine proteases activation

**Fig 4:**
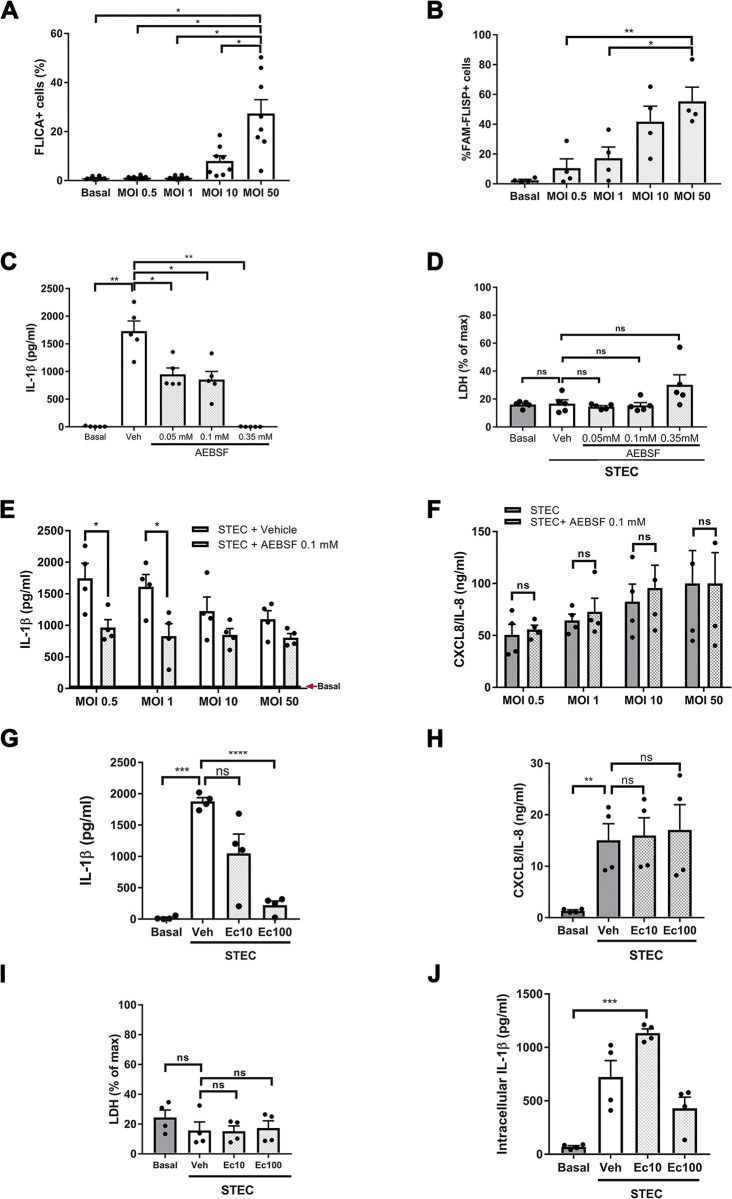
Neutrophil serine proteases (NSPs) and caspase-1 are required for IL-1β secretion upon challenge with STEC. (**A and B**) Neutrophils were challenged with STEC at the MOI indicated for 2 h and then stained with the fluorescent probe FLICA to detect active caspase-1 (**A**) or with the fluorescent probe FAM-FLISP to detect active serine proteases (**B**). Then, the percentage of positive cells were determined by flow cytometry. (**C-F**) Neutrophils were treated with vehicle (Veh) or with the serine proteases inhibitor AEBSF at the indicated concentrations for 30 min and then challenged or not (basal) with STEC at a MOI of 0.5 (**C** and **D**) or 0.5–50 (**E** and **F**) for 3h at 37°C. (**G-J**) Neutrophils were treated with Veh or with the human elastase inhibitor Ecotin (10 or 100 μg/ml) for 30 min and then challenged or not (basal) with STEC at a MOI of 0.5 for 3 h at 37°C. Supernatants were collected and IL-1β (**C, E** and **G**) and CXCL8/IL-8 (**F** and **H**) concentration were determined by ELISA, or LDH levels (**D** and **I**) were examined by a cytotoxicity assay kit. (**J**) Intracellular IL-1β levels were assessed in the cell pellets of experiments shown in **G** by ELISA. Graphs depict the mean ± SEM of experiments performed in triplicate; each dot represents the triplicate´s mean for each individual donor sample. Statistical significance between samples was assessed by One-way ANOVA followed by Sidak´s multiple comparisons test for data with normal distribution and Friedman test followed by Dunn’s multiple comparisons test for data with non-normal distribution. *p<0.05; **p<0.01; ***p<0.001; ****p<0.0001; ns: non-significant.

To get insight into the mechanisms responsible for the reduction in IL-1β secretion as STEC MOI increase, we further evaluated caspase-1 activation in neutrophils challenged with STEC at different MOI, by employing the FLICA fluorescent probe. We found significant activation of the enzyme at a MOI of 50, even though a trend to an increased percentage of FLICA+ cells was also detected at a MOI of 10 (Fig 4A). However, these findings also suggest the probe was not sensitive enough to detect caspase-1 activation at lower MOI, because in Fig 2B and 2C, the caspase-1 specific inhibitors Ac-YVAD-CMYK and VX-765, practically abolished IL-1β secretion by neutrophils challenged with STEC at MOI 0.5, confirming the participation of the enzyme even at a low MOI.

In a previous study, we found that IL-1β secretion by human neutrophils stimulated with LPS+ATP is dependent not only on caspase-1 but also on the NSPs activity which are able to leak from azurophil granules to the cytosol and process pro-IL-1β [17]. Thus, we then evaluated NSPs activation by STEC by employing a fluorescent probe that stains active chymotrypsin-like serine proteases, among them NSPs. As shown in Fig 4B, a significant rise in the percentage of FAM-FLISP-positive cells was detected as the MOI increased. Furthermore, we found that pre-treatment of neutrophils with AEBSF, an irreversible PAN serine protease inhibitor, reduced neutrophil IL-1β secretion in a concentration-dependent fashion (Fig 4C) and did not induce neutrophil lytic death (Fig 4D). Moreover, AEBSF was able to significantly reduce IL-1β secretion at the lower MOI (MOI 0.5 and 1; Fig 4E) while it did not modulate CXCL8/IL-8 secretion (Fig 4F). Of note, the levels of IL-1β released by cells treated with AEBSF were similar at all the MOI evaluated, suggesting that differences observed in the absence of the inhibitor at the distinct MOI were a consequence of the activity of NSPs.

To get further evidence that the inhibition of NSPs is responsible for the reduction of STEC-induced IL-1β secretion, and rule out potential off-target effects that might exert AEBSF, we performed additional assays by employing Ecotin, a serine protease inhibitor [20,21], which was able to reduce human neutrophil elastase (NE) activity (S6 Fig). As shown in Fig 4G, Ecotin also inhibited IL-1β secretion in a concentration-dependent manner but neither affected CXCL8/IL-8 secretion (Fig 4H) nor induced neutrophil lytic death (Fig 4I). Moreover, at the lower Ecotin concentration (10 μg/ml), but not at the higher (100 μg/ml), we detected some mature IL-1β inside the cells, suggesting that some NSPs remained active, being able to process pro-IL-1β just as caspase-1 did (Fig 4J).

### IL-1β secretion depends on GSDMD pores which control NSPs activity and consequently pro-IL-1β processing or eventually its degradation

**Fig 5:**
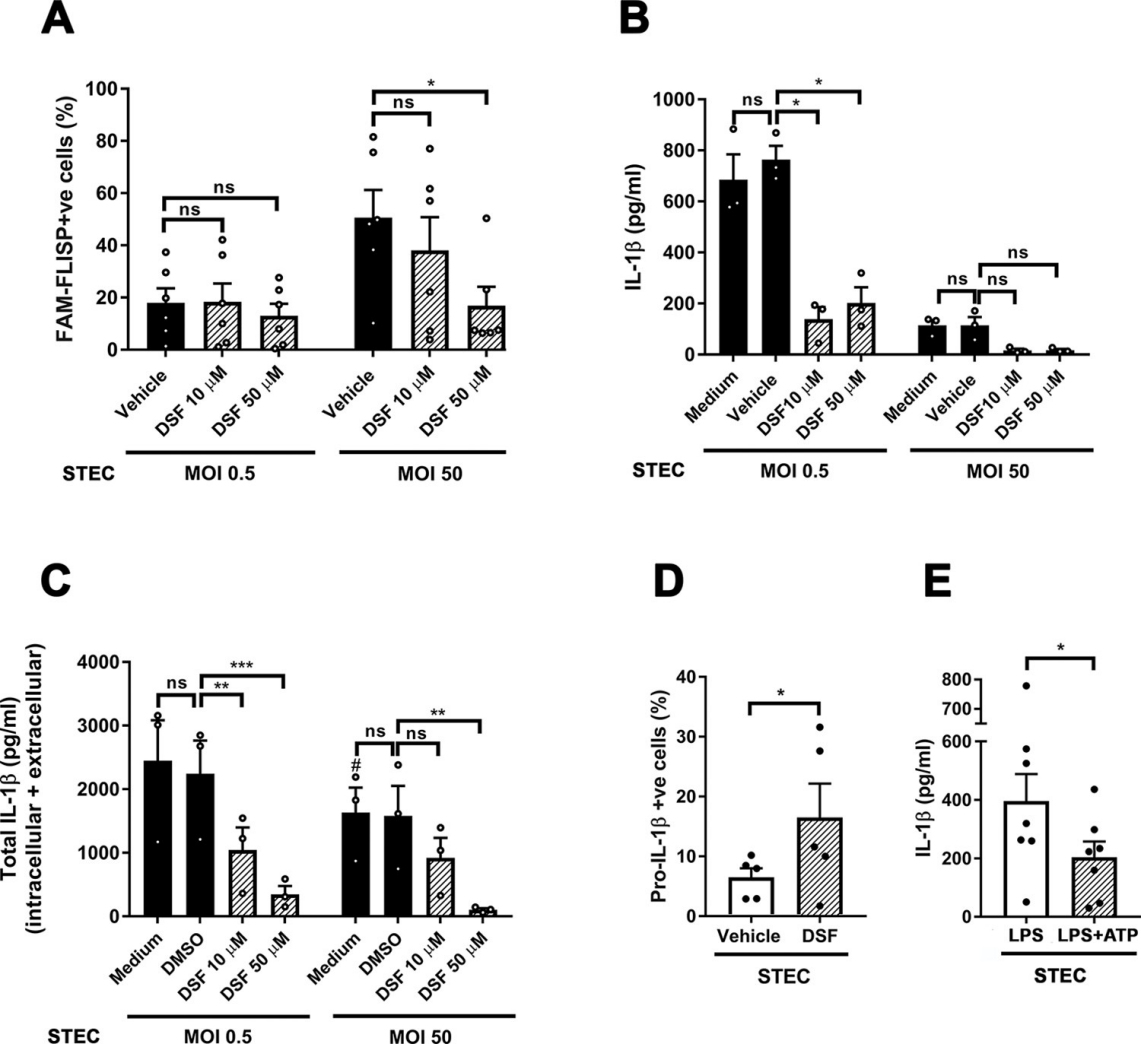
The GSDMD pores control the NSPs activity and consequently the pro-IL-1β processing or degradation. (**A-D**) Neutrophils were pretreated or not with DSF for 30 min, and then challenged with STEC at MOI 0.5 (**A-E**) and MOI 50 (**A-C**). Two hours later they were stained with the FAM-FLISP probe and the percentage of cells with active NSPs was determined by flow cytometry (**A**); or 3.5 h later (**B and C**) the intracellular and extracellular IL-1β concentrations were determined by ELISA, or (**D**) 2.45 h later the intracellular pro-IL-1β levels were determined by immunostaining and flow cytometry. Graphs depict (**B**) IL-1β concentrations in culture supernatants; (**C**) total (intracellular + extracellular) IL-1β concentrations, and (**D**) the percentage of pro-IL-1β positive cells. (**E**) Neutrophils were stimulated with LPS (150 ng/ml) for 2 h, then were treated with vehicle or ATP and 30 min later were washed and challenged with STEC (MOI 0.5) and 3 h later the IL-1β concentrations in culture supernatants were determined by ELISA. (**B**, **C** and **E**) Graphs depict the mean ± SEM of experiments performed in triplicate; each dot represents the triplicate´s mean for each individual donor sample. Statistical significance between samples was assessed by One-way ANOVA followed by Sidak´s multiple comparisons test for data with normal distribution and Friedman test followed by Dunn’s multiple comparisons test for data with non-normal distribution, except in D and E in which data were analyzed by t test. *p<0.05; **p<0.01; ***p<0.001; ns: non-significant.

Previous studies showed that active GSDMD produced by caspase-1 processing can form pores on the membranes of neutrophil azurophil granules that allow the leakage of NE to the cytosol, which in turn, can drive a secondary phase of GSDMD proteolytic processing [22,23]. Thus, a stronger activation of caspase-1 might induce larger NE leakage to the cytosol. Considering that the IL-1β sequence contains many potential NE cleavage sites, a huge amount of active NSPs instead of contributing to IL-1β processing might lead to its degradation. On this basis, we here hypothesized that the reduction in IL-1β secretion observed when neutrophils were challenged with STEC at higher MOI could be due, at least in part, to the higher activation of both caspase-1 and NSPs triggered in those conditions (Fig 4A and 4B). We reasoned that for this being a possibility, inhibition of GSDMD oligomerization would lead to both reductions in NSPs activation and IL-1β secretion. To test this hypothesis, we evaluated the effect of Disulfiram (DSF), an inhibitor of GSDMD pore formation [24]. DSF significantly reduced the strong activation of NSPs induced by STEC when employed at MOI 50 (Fig 5A) in agreement with a role of the GSDMD pore in the release of NSPs to the cytosol and their consequent activation. DSF also showed a trend to reduce the slight NSPs activation observed at MOI 0.5 (Fig 5A). Moreover, DSF also markedly inhibited IL-1β secretion (Fig 5B). In accordance with a role of the NSPs leaked from granules through the GSDMD pore in pro-IL-1β processing/degradation, DSF also reduced the total levels (intracellular+extracellular) of mature IL-1β (Fig 5C), and led to an increase in the intracellular pro-IL-1β levels (Fig 5D). In line with our hypothesis, pre-activation of caspase-1 by treatment of neutrophils with LPS+ATP led to a marked reduction in IL-1β secretion when cells were then challenged with STEC as compared to cells that had not been pretreated with ATP (Fig 5E). Of mention, none of these treatments modulated neutrophil lytic death (S7A and S7B Fig). Altogether, these results are in accordance with the notion that the higher the STEC MOI that neutrophils face, the higher the NSPs leakage to the cytosol, which instead of contributing to IL-1β processing can lead to IL-1β degradation.

### IL-1β secretion is also regulated by the intensity of the oxidative burst which controls the activity of NSPs

**Fig 6:**
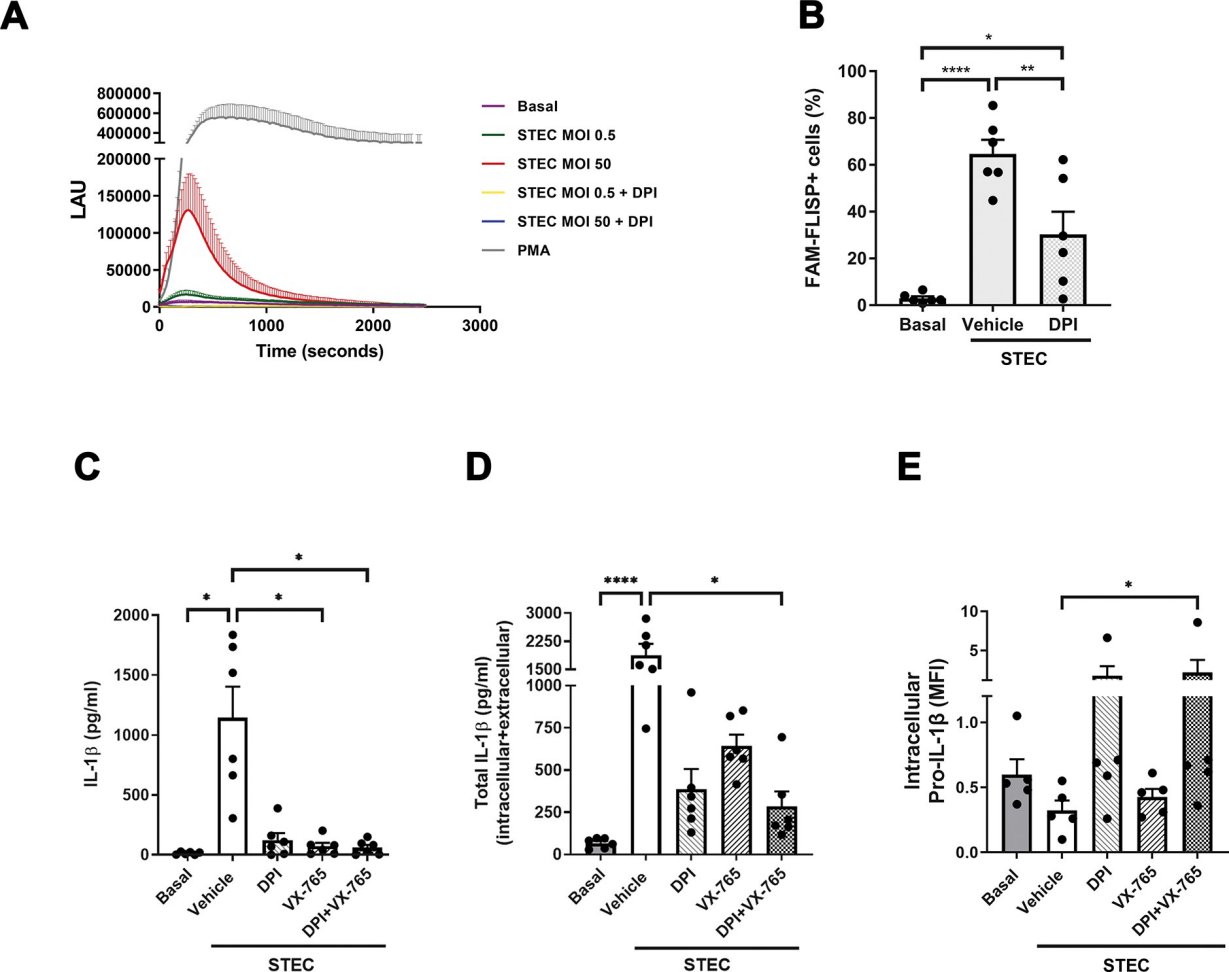
ROS produced by NADPH oxidase in response to STEC control NSPs activity and consequently pro-IL-1β processing or degradation. (**A**) Neutrophils were pretreated or not with the NADPH oxidase inhibitor DPI (10 μM) for 30 min, and then challenged or not with STEC at the indicated MOI, or with PMA (50 ng/ml) as positive control; and the kinetic of the ROS response was determined with a luminol-based luminescence assay in a microplate reader. Measurements were performed every 20 seconds. The succession of points in each line represents the mean ± SEM values of 3 experiments performed with neutrophils of different donors. (**B**) Neutrophils were pretreated or not with DPI (10 μM) for 30 min, and then challenged or not with STEC at MOI 50 for 2 h. Then, they were stained with the FAM-FLISP probe and the percentage of cells with active NSPs was determined by flow cytometry. (**C-E**) Neutrophils were pretreated or not with DPI (10 μM), VX-765 (50 μM) or both inhibitors together for 30 min, and then challenged with STEC at a MOI of 0.5 for 3 h. Then, the extracellular (**C**) and the total (intracellular+extracellular) (**D**) IL-1β levels were determined by ELISA; and the pro-IL-1β levels (**E**) were determined by intracellular immunostaining and flow cytometry. (**C-D**) Graphs depict the mean ± SEM of experiments performed in triplicate; each dot represents the triplicate´s mean for each individual donor sample. Statistical significance between samples was assessed by One-way ANOVA followed by Sidak´s multiple comparisons test for data with normal distribution and Friedman test followed by Dunn’s multiple comparisons test for data with non-normal distribution. *p<0.05; **p<0.01; ****p<0.0001.

A previous study determined that leakage of NE from azurophil granules to the cytosol can also be mediated by reactive oxygen species (ROS) which trigger the dissociation of NE from the azurosome (a complex located in the azurophil granule membranes) into the cytosol, and the activation of its proteolytic function [25]. Thus, we then investigated if differences in the levels of IL-1β secretion observed when neutrophils were challenged with STEC at distinct MOI could also be determined by differences in the intensity of the oxidative burst. As shown in Fig 6A, STEC induced a strong ROS response when neutrophils were challenged at MOI 50 (red line) and a very weak response at MOI 0.5 (green line). Both responses were abrogated by the NADPH oxidase inhibitor Diphenyleneiodonium chloride (DPI; yellow and blue lines). As expected, in accordance with a role of ROS in the activation of serine proteases, the inhibition of NADPH oxidase significantly reduced the percentage of cells with active NSPs (Fig 6B). Moreover, DPI also tended to reduce IL-1β secretion (Fig 6C) and to diminish the level of total (intracellular+extracellular) mature IL-1β (Fig 6D) induced by STEC, which also agrees with a role of NSPs in pro-IL-1β processing. Treatment of neutrophils with VX-765 significantly reduced extracellular IL-1β levels (Fig 6C) and, consistent with our previous data indicating that inhibition of caspase-1 does not inhibit pro-IL-1β processing [17], it did not significantly modulate intracellular mature IL-1β levels (Fig 6D). We then reasoned that these results might be because when caspase-1 is inhibited, cytosolic NSPs released from azurophil granules by the ROS-mediated mechanism were still able to process pro-IL-1β. Thus, treatment with VX-765 together with DPI, which would block NSPs leakage to the cytosol, should reduce IL-1β processing and secretion. Supporting our hypothesis, both inhibitors together abrogated IL-1β release (Fig 6C) and impaired the cytokine processing, because either the total levels of mature IL-1β were significantly reduced (Fig 6D), and the intracellular pro-IL-1β levels were significantly increased (Fig 6E). Of mention, treatments with the inhibitors did not modulate neutrophil lytic death (S7C Fig).

These findings together with those in Fig 5 suggest that the intensity of the neutrophil oxidative response and the levels of caspase-1 activation upon challenge with STEC at different MOI determine if pro-IL-1β is processed to its active isoform and secreted or is intracellularly degraded.

### Pharmacologic inhibition of serine proteases does not affect the STEC killing by neutrophils

**Fig 7:**
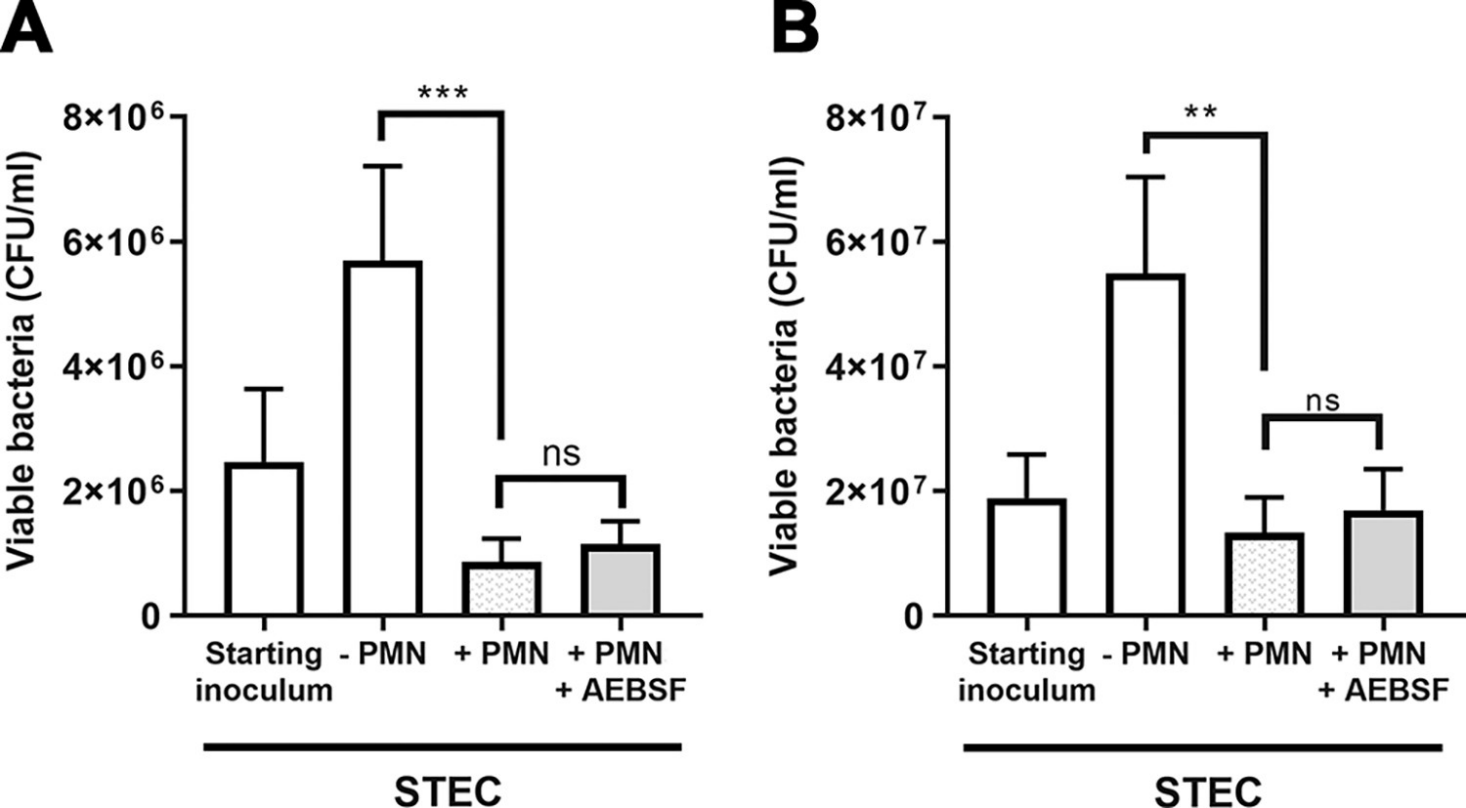
Inhibition of NSPs does not affect the capacity of neutrophils to kill STEC. Neutrophils were treated or not with AEBSF (0.1 mM) for 30 min, and then challenged with STEC (A; MOI = 1; B, MOI = 10) for 1 h at 37°C. DNase I (10 U/mL) was added in the last 15 min of culture. Then, supernatants were collected, the cell pellets were lysed and mixed with the supernatant fractions. The viable bacteria in each sample were quantified by plating out serial dilutions of them on TSA medium, incubation for 18 h at 37°C and 5% CO_2_ and CFU counting. The starting inoculum represents the number of CFU present at the beginning of the experiment. The “-PMN” condition represents the CFU after 1 h of culture in the absence of neutrophils, while the conditions +PMN represent those in which bacteria where co-incubated for 1 h with neutrophils either pretreated (+AEBSF) or not with this inhibitor. Data are depicted as the mean ± SEM of 4 assays. Statistical significance between samples was assessed by Friedman test followed by Dunn’s multiple comparisons test. **p<0.01; ***p<0.001; ns: non-significant.

Finally, we performed additional assays to examine if it would be worth considering NSPs as a potential therapeutic target to reduce STEC-induced inflammation. We reasoned that for this being a possibility, NSPs inhibition should not impede the ability of neutrophils to kill the bacteria, mainly considering that antibiotics are not therapeutic options in STEC infections [26,27]. To evaluate this fact, we pretreated or not neutrophils with AEBSF and challenged them with STEC, and one hour later determined the number of viable bacteria. Our results indicated that inhibition of NSPs did not affect the STEC killing capacity of human neutrophils (Fig 7A and 7B).

## Discussion

A growing body of evidence shows the relevance of neutrophil secreted IL-1β in immune responses against different pathogens, especially in murine models of infection [28–34]. However, the ability of neutrophils to secrete IL-1β in response to the highly pathogenic STEC O157:H7 has not been previously addressed. This fact becomes relevant since upon STEC infections, neutrophils infiltrate the intestinal mucosa and the enterohaemorrhagic *E*. *coli*-induced gastroenteritis is accompanied by marked inflammation and mucosal damage [7]. Thus, neutrophils not only might contribute to bacterial removal but also to mucosal damage and HUS development by secreting IL-1β. This cytokine might lead both to additional neutrophil recruitment that would facilitate Stx access to the circulation [9] and to sensitize the endothelium to Stx action by increasing its receptor, the globotriaosylceramide receptor (Gb3) expression [35].

Results of our study show that upon STEC challenge, human neutrophils secrete high amounts of IL-1β, especially when faced with bacteria at low MOI. This situation probably takes place at the early time points after infection because STEC shows a formidable ability to establish infections at much lower doses than for most of intestinal pathogens [19]. Our findings also indicated that neutrophil IL-1β secretion induced by STEC does not depend on the ability of the bacteria to produce Stx because 1) an isogenic mutant lacking the ability to produce the toxin (ΔSTEC strain) induced similar neutrophil IL-1β secretion levels; 2) the replacement of the supernatant of a ΔSTEC culture by a STEC supernatant did not affect the cytokine secretion by neutrophils, as neither did 3) the addition of purified Stx2 to a ΔSTEC culture before challenging neutrophils; and 4) bacterial supernatant containing Stx did not induce neutrophil IL-1β secretion. These findings agree with the fact that neutrophils do not express Gb3. Nevertheless, these cells can recognize Stx by TLR4, the LPS receptor. However, as previous studies showed that LPS compete with Stx for TLR4 binding [36], probably LPS present in the whole bacteria impairs Stx interaction with this receptor, explaining the absence of a role for this toxin in neutrophil IL-1β secretion. Yet, one or more bacterial pathogenicity factors appear to be involved in the STEC-induced neutrophil IL-1β response as a non-pathogenic *E*. *coli* strain did not induce significant IL-1β levels. A previous study showed that *E*. *coli* O157:H7-enterohemolysin (Ehx) is required for IL-1β secretion in THP-1 cells [37]. However, this study also found that Ehx contributed to THP-1 cell toxicity and showed a positive correlation between IL-1β secretion and LDH release to the supernatants upon infection with Ehx-expressing bacteria. Since we did not observe an increase in LDH levels in supernatants upon the challenge of neutrophils with STEC even at different MOI, and the fact that bacterial supernatants were unable to induce IL-1β secretion, Ehx is unlikely to be involved in neutrophil IL-1β release. Furthermore, our findings also indicated that bacterial viability is required to stimulate neutrophil IL-1β secretion, suggesting that an active mechanism exerted by bacteria is involved. However, the identity of this mechanism remains to be determined. It is tempting to speculate that the STEC type III secretion system (T3SS) is implicated since our results indicated that NLRP3 is involved in IL-1β secretion, and previous studies showed that *Salmonella enterica* induces NLRP3 activation upon THP-1 infection associated with the activity of the T3SS [38].

Our results also indicated that neutrophil IL-1β secretion triggered by STEC significantly decreased as MOI increased. This behavior was particular for this cytokine as CXCL8/IL-8 secretion did not show the same profile. Moreover, it was neither associated with differences in neutrophil death levels at different MOI nor with an increase in Stx levels at high MOI because the same profile was observed when granulocytes were challenged with ΔSTEC. Furthermore, we found that both caspase-1 and NSPs are required for neutrophil IL-1β secretion, as caspase-1 specific inhibitors (Ac-YVAD-CMK and VX-765) and serin proteases inhibitors (AEBSF and Ecotin) markedly reduced IL-1β release. In previous studies, we found that the secretion of this cytokine in response to LPS+ATP also involves both kinds of enzymes and described an interplay between them that regulates pro-IL-1β processing and IL-1β release [17]. In fact, in that study, we showed that NSPs can contribute both to inactivating caspase-1 and to processing pro-IL-1β but are also potentially capable to degrade the cytokine. Furthermore, we showed that NSPs, but not caspase-1, accomplish a major role in pro-IL-1β processing [17]. Results in our current study both confirm that this interplay between caspase-1 and NSPs also takes place when neutrophils are challenged with a bacterium and incorporate the NADPH oxidase-derived ROS as additional players in this regulatory mechanism. In fact, we found that the greater the activity of both caspase-1 and NSPs, the lower the release of IL-1β, even though both enzymes were required for this cytokine release. As previously mentioned, Karmakar et al. showed that neutrophils’ caspase-1 activation leads to GSDMD cleavage generating an N-terminal GSDMD product that mainly associates with azurophil granule membranes forming a pore that allows the leakage of elastase to the cytosol and its activation [22]. In line with these findings, we here found that DSF, a compound that blocks GSDMD pore formation, inhibited the activation NSPs induced by STEC. Moreover, DSF also reduced pro-IL-1β processing as judged by the reduction of both the IL-1β secretion and the total levels (intracellular+extracellular) of mature IL-1β, and the increment in pro-IL-1β levels. Thus, together, our results suggest that when neutrophils were challenged with STEC at low MOI, the reduced activation of caspase-1 led to minor leakage of NSPs to the cytosol, and both enzymes probably contributed to pro-IL-1β processing. However, when neutrophils were challenged with STEC at high MOI, the strong activation of caspase-1 induced a huge release of NSPs to the cytosol, which instead of contributing to pro-IL-1β processing, led to the cytokine degradation. In agreement with this possibility, we found that neutrophils pretreated with LPS+ATP to activate caspase-1 before being challenged with STEC, released lower IL-1β levels compared to those secreted by non-ATP-pre-treated cells.

**Fig 8:**
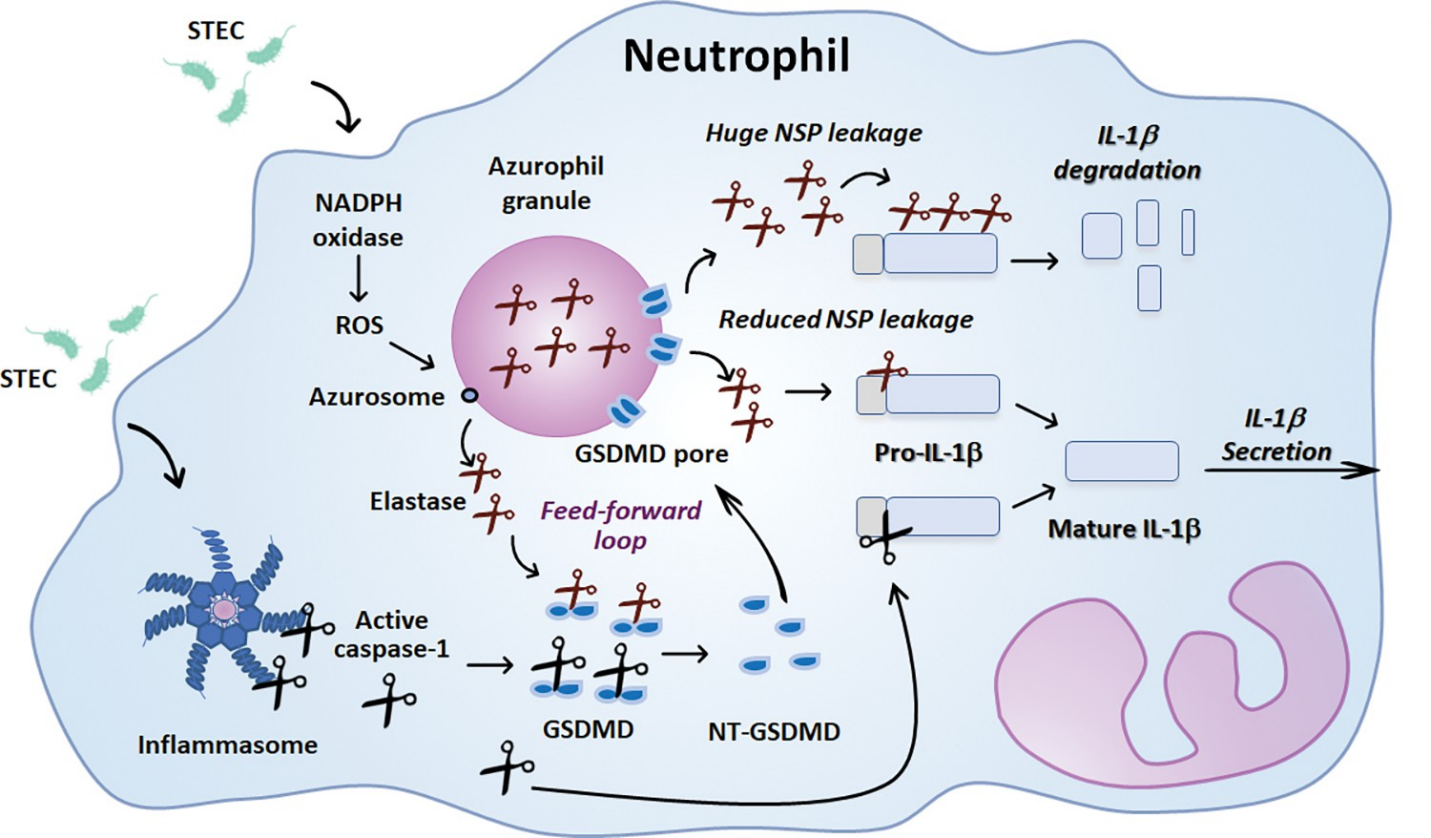
Graphical model. **https://openclipart.org/detail/36289/scissors**. Challenge of neutrophils with STEC induces ROS production dependent on NADPH oxidase activation. ROS would trigger elastase release from azurophil granules to the cytosol which could be able to process both Gasdermin D (GSDMD) and pro-IL-1β. Likewise, STEC also trigger inflammasome activation and consequently caspase-1 activation which is able to also process both GSDMD and pro-IL-1β. The N-terminal fragment of GSDMD can form pores on azurophil granule membranes increasing elastase leakage to the cytosol. The amount of active cytosolic elastase would determine if pro-IL-1β is processed to the mature IL-1β isoform or degraded.

On the other hand, Metzler et al. revealed that ROS produced by neutrophils in response to PMA or Candida induce the release from the azurosome (a complex located in the azurophil granule membranes) into the cytosol of NE and cathepsin G and activate their proteolytic activity [25]. A more recent study by Zychlinsky´s group showed that cytosolic NE cleaves GSDMD, and the processed-GSDMD in turn, forms pores in the granule membrane, thus enhancing NE release into the cytoplasm, which allows further GSDMD cleavage in an iterative process [23]. Noteworthy, the authors also found that neutrophils from patients with X-linked chronic granulomatous disease (CGD) incapable to produce ROS from NADPH oxidase were also unable to cleave GSDMD upon stimulation with PMA. In this study, we found that higher STEC MOI induce stronger ROS responses and greater NSPs-activity. We also found that the NADPH oxidase inhibitor reduced the percentage of cells with active NSPs and tended to diminish IL-1β secretion and to increase pro-IL-1β levels. But noteworthy, when the NADPH oxidase and the caspase-1 were simultaneously inhibited, both IL-1β secretion and pro-IL-1β processing were nearly abrogated, and a significant accumulation of pro-IL-1β could be intracellularly detected. Thus, these results suggest that an increased activity of cytosolic NSPs initially released from the azurosomes by a ROS-dependent mechanism also contributes to the decreased release of IL-1β observed at higher STEC MOI, because instead of leading to IL-1β processing can contribute to its degradation. Altogether, the results of this study support that pro-IL-1β processing and IL-1β release in response to STEC are regulated by an interplay between NSPs and caspase-1, in which the NADPH oxidase derived-ROS also participate (Fig 8).

Considering that STEC infections usually occur at low infective doses, it is dared to speculate that at the beginning of the infection, neutrophils recruited to the gut secrete high IL-1β levels through a mechanism that involves caspase-1 and NSPs activity. However, as bacterial replication progresses, stimulation of neutrophils at high MOI would induce higher ROS levels, and more NSPs leakage to the cytosol that instead of contributing to pro-IL-1β processing would lead to its degradation; and according to our previous studies with LPS+ATP, probably also to caspase-1 inactivation. Furthermore, NE might promote additional NSPs leakage to the cytosol, in a feed-forward loop as has been previously proposed in cells stimulated with phorbol ester [23].

Our findings indicating the interplay between the activities of NSPs and caspase-1 regulates IL-1β secretion become relevant not only in relation to *E*. *coli* infections but also regarding human neutrophil proinflammatory capacity, because together with our previous results with LPS+ATP, it shows that a common mechanism controls the intensity of IL-1β response avoiding the excessive release of this extremely proinflammatory cytokine.

Finally, considering that NSPs partial inhibition reduced IL-1β secretion but did not affect the neutrophil STEC killing capacity, our findings also pave the way to investigate the therapeutic potential of inhibiting NSPs to reduce inflammation associated with STEC infections and in turn the progression to HUS.

## Materials and methods

### Ethics statement

The experimental protocols performed have been approved by the Biosafety and Research Review boards of the “Instituto de Medicina Experimental (IMEX)-CONICET-Academia Nacional de Medicina” and the Ethical Committee of the “Institutos de la Academia Nacional de Medicina”. Approval numbers T.I. 12843/18/X and T.I. 13065/19/X. Blood samples were collected from volunteer healthy donors who gave written consent.

### Reagents and materials

RPMI 1640 no phenol red culture medium, CyQUANT LDH Cytotoxicity Assay Kit, dextran 500, and TMB substrate were purchased from Thermo Fisher Scientific Life Technologies (MA, USA). Fetal bovine serum (FBS) and bovine serum albumin were purchased from Internegocios (Buenos Aires, Argentina). Ficoll PM400 was purchased from Cytiva (Uppsala, Sweden). Triyosom 50 was obtained from Gobbi Novag S.A. (Buenos Aires, Argentina). BD OptEIA Human IL-1β ELISA Set II and Human IL-8/CXCL8 ELISA Set were purchased from BD Biosciences (Franklin Lakes, NJ, USA). Secondary antibodies were purchased from Jackson Immunoresearch Laboratories (West Grove, PA, USA): Alexa Fluor 488 AffiniPure F(ab’)2 Fragment Goat Anti-Rabbit IgG (H + L) cat. #111-546-144;. TO-PRO-3 was obtained from Life Technologies (Carlsbad, CA, USA). Phycoerythrin-conjugated anti-CD14 antibody was purchased from eBioscience (San Diego, CA, USA). Aqua Poly/Mount Coverslipping Medium was purchased from Polysciences (Warrington, PA, USA). Rabbit polyclonal anti-human IL-1 beta cat. NB600-633 was from Novus Biologicals (Co, USA); rabbit polyclonal anti-human-myeloperoxidase cat.#A0398 was from Dako (Glostrup, Denmark); and purified mouse IgG1, κ isotype control cat. #555746 and rabbit polyclonal IgG was purchased from Jackson Immunoresearch. Complete, EDTA-free Protease Inhibitor Cocktail, cat. #11873580001 was from Roche (Basel, Switzerland). AEBSF (4-(2-aminoethyl)-benzenesulfonyl fluoride, monohydrochloride), Ac-YVAD-CMK (N-acetyl-L-tyrosyl-L-valyl-N-[(1S)-1-(carboxymethyl)-3-chloro-2-oxo-propyl]-L-alaninamide), Z-VAD-FMK, MCC950, VX-765 and Elastase fluorescent substrate ((Z-Ala-Ala-Ala-Ala)2Rh110) were purchased from Cayman Chemical (Michigan, USA). FAM-FLICA Caspase-1 Assay Kit and FAM-FLISP FLCK Serine protease kit were purchased from Immunochemistry Technologies (Bloomington, MN, USA). Human TruStain FcX was purchased from Biolegend (San Diego, USA). Recombinant Ecotin from *Salmonella* Typhimurium was obtained by expression and purification in *E*. *coli* using a 6xHis-tag. Tryptic soy broth (TSB) was purchased from Neogen (Michigan, USA). All other chemicals employed were purchased from Sigma Aldrich (St. Louis, MO, USA).

### Human neutrophil isolation

Cells were isolated from ACD-anticoagulated human blood from healthy adult donors who have given written consent. Neutrophils were isolated by centrifugation on Ficoll-Triyosom, followed by dextran sedimentation, and hypotonic lysis. Cells were suspended at 6 × 10^6^/mL in RPMI 1640 without phenol red and 10% FBS. After purification, neutrophil preparations were stained with an anti-CD14-PE antibody and analyzed by flow cytometry to guarantee that monocyte contamination was less than 0.5%. Cells were used immediately after isolation.

### Bacterial strains and growth conditions

The bacterial strains used in this study were *Escherichia coli* (*E*. *coli*) serotype O157:H7 strain 125/99 (STEC); *E*. *coli* O157:H7 devoid of the capacity to produce Stx (ΔSTEC) [18,39] and the *E*. *coli* C600 strain. The *E*. *coli* serotype O157:H7 strain 125/99 was isolated from fecal specimens of a patient with HUS [40]. This strain belonged to the serotype O157:H7, and harbored the *eae, ehxA* and *stx2a* genes; but not *stx1* [41]. The ΔSTEC strain was kindly provided by Dr. Angel Cataldi (IABIMO-CONICET, INTA-Castelar, Buenos Aires, Argentina).

All bacterial strains were cultured overnight in RPMI 1640 without phenol red medium with 0.05% NH_4_Cl and 2% glucose (bacterial growth medium–BGM-) at 37°C with shaking at 200 rpm. To quantify the bacteria for calculating the multiplicity of infection (MOI), serial dilutions were seeded on tryptic soy agar (TSA) plates, and colonies-forming units (CFU) were counted. CFU/ml were estimated as the average number of CFU counted in at least six replicate plates.

Bacterial supernatants were collected after centrifugation at 9600 × g for 10 min.

The ability of STEC to produce toxin when growing in BGM (S8 Fig) was determined through a Stx-cytotoxicity bioassay with Vero Cells by comparison of the results with those obtained in parallel with STEC grown in tryptic soy broth (TSB), as described below.

Heat-killed bacteria were obtained by incubation at 60°C for 1 h. Bacterial death was confirmed through serial dilutions seeding on TSA plates.

### Stx cytotoxicity bioassay with Vero Cells

The Stx2-sensitive Vero cell line was grown in RPMI 1640 medium supplemented with 10% FBS, penicillin, and streptomycin on 96-well plates for 18 h at 37°C.

STEC and ΔSTEC were cultured overnight in BGM or TSB medium at 37°C with shaking at 200 rpm. By the end of the incubation, an Optical Density (OD) at 600 nm of 0.9 was reached, and the corresponding CFU/ml were estimated. Cultures were centrifuged for 10 minutes at 9600 x g and serial dilutions of the supernatants were seeded on Vero cells monolayers previously grown in 96-well plates and incubated for 48 h at 37°C and 5% CO_2_ for further assessment of Stx presence. Afterward cells were washed with PBS, then fixed and stained for 5 min at room temperature (RT) with 0.1% crystal violet with 20% methanol. Then, wells were washed thrice with fresh water and the remaining cells were solubilized using 30% acetic acid for 20 min at RT. Absorbance at 550 nm was determined in a spectrophotometer as a measurement of cell viability. The comparison of the cytotoxic capacity of culture supernatants of STEC and ΔSTEC on Vero cells is shown in S9 Fig.

### Neutrophil culture and stimulation

Neutrophils (5.4x10^6^/ml) were challenged or not with live- or heat-killed STEC, ΔSTEC, or *E*. *coli* C600, or stimulated or not with the bacterial supernatants for the specified times in each experiment at 37°C and 5% CO_2_. In some experiments before bacterial challenge, neutrophils were pretreated for 30 min with AEBSF (0.1 mM), Ac-YVAD-CMK (50 μM), VX-765 (50 μM), Z-VAD-FMK (50 μM), MCC950 (10 μM), DPI (10 μM), Ecotin (10 or 100 μM), DPI (10 μM) + VX-765 (50 μM), or ATP (2.5 mM). After culture, the supernatants, and in the indicated assays, the cell pellets were collected, and IL-1β and CXCL8/IL-8 concentrations were quantitated by ELISA. Neutrophil lytic death was determined by evaluation of the lactate dehydrogenase (LDH) activity in cell culture supernatants. Alternatively, at the specified times, cell pellets were fixed with 4% paraformaldehyde (PFA) and processed for either confocal laser scanning microscopy (CLSM) or flow cytometry.

### LDH assay

The LDH levels in the supernatants were determined using the CyQUANT LDH Cytotoxicity Assay Kit, following the manufacturer’s instructions. The samples were centrifuged and then supernatants were collected for further evaluation. The absorbance of the samples was measured with a spectrophotometer at wavelengths of 490 nm and 680 nm (background). The LDH activity was calculated by subtracting the absorbance value at 680 nm from the absorbance value at 490 nm. The enzyme activity was expressed as a percentage of the maximum value obtained from an equivalent number of neutrophils lysed with 0.1% Triton X-100.

### Cytokine quantification by ELISA

Supernatants of neutrophils challenged with bacteria for 3.5 h at 37°C and 5% CO_2_ were collected and the concentrations of human IL-1β and human CXCL8/IL-8 were quantified by ELISA following the manufacturer’s instructions.

### Neutrophil serine proteases (NSPs) activation assessment

Neutrophils (5.4 x 10^5^) were challenged with STEC at increasing MOI (0.5–50) at 37°C and 5% CO_2_. After 1 h 45 min, the fluorescent NSPs probe FAM-FLISP FLCK was added following the manufacturer’s instructions, and 15 min later samples were washed, fixed and their fluorescence was determined by flow cytometry.

### Caspase-1 activation assessment

Neutrophils were incubated with FAM-FLICA Caspase-1 (YVAD) reagent (FLICA) according to the manufacturer’s instructions for five minutes before bacterial stimulation. After 40 min of culture, cells were washed thoroughly, fixed with PFA 4% and fluorescence was determined by flow cytometry.

### Flow cytometry analysis

For certain experiments, after immunostaining, cell fluorescence was determined by flow cytometry using a Partec Cyflow cytometer. Data were analyzed by using the FlowJo software (FlowJo v10.3 for Windows; Treestar Inc, Ashland, OR, USA).

### Killing assay

Neutrophils were challenged with STEC at MOI 1 or 10 and co-cultured for 1 h at 37°C and 5% of CO_2_. In the last 15 min of culture, DNase I (10 U/mL) was added to release bacteria potentially entrapped in NETs. Samples were centrifuged, the supernatants were collected, and the cell pellets were lysed with distilled water and mixed with the supernatant fractions. These preparations were then plated in sextuplicate in TSA medium. Plates were incubated at 37°C for 18 h, and then the number of CFU were counted.

### Reactive oxygen species (ROS) detection assay

Neutrophils were incubated with or without STEC at MOI of 0.5 and 50, or PMA (50 ng/ml) as a positive control, and supplemented with luminol (0.1 mM). Chemiluminescence were measured immediately after the addition of luminol (5-amino-2, 3-dihydro-1, 4-phthalazinedione) in a Varioskan-Lux plate reader for 45 minutes at 37°C.

### Intracellular immunostainings

After fixation with PFA 4% for 30 min, cells were blocked with PBS-glycine (0.1 M) for 15 min, permeabilized with chilled acetone (−20°C) for 7 min, rehydrated with PBS and blocked with PBS supplemented with 5% goat serum overnight at 4°C. Then, neutrophils were incubated with the Fc Receptor Blocking Solution (Human TruStain FcX) for 5 minutes, then with the primary antibodies in blocking buffer for 1 h at room temperature, washed, and then incubated with the corresponding secondary antibody for 1 h at room temperature. Cells were analyzed by flow cytometry.

### Quantification of cells with lobulated nuclei

Neutrophils were cultured for 3.5 h at 37°C and 5% CO_2_ without (basal) or with STEC at MOI 0.5 or MOI 50. Then, cells were fixed with 4% PFA and stained with propidium iodide and images were captured by using a FluoView FV1000 confocal microscope (Olympus, Tokyo, Japan) equipped with a Plapon 60X/1.42 objective. Analysis was performed by determining the number of neutrophils with lobulated nuclei per microscopic field.

### Statistical analysis

Statistical analysis was performed using GraphPad Prism v7.00 for Windows, GraphPad Software, La Jolla, CA, USA. Data were analyzed for normality using the Shapiro-Wilk normality test. Statistical analyses on normally distributed data sets were performed using One-way ANOVA followed by Sidak´s multiple comparisons test, and where indicated, Two-way ANOVA followed by Sidak´s multiple comparisons test and Tukey’s multiple comparisons test. Data sets with non-normal distributions were analyzed using the nonparametric Friedman´s test followed by Dunn’s multiple comparisons test. Statistical significance was defined as *p<0.05; **p<0.01; ***p<0.001; ****p<0.0001; ns: non-significant.

## Supporting information

S1 Data Numerical data used in all figures. Excel spreadsheet containing, on separate sheets, the underlying numerical data and statistical analysis for all the figures in this work.
(XLSX)

S1 Fig STEC and ΔSTEC do not induce neutrophil lytic death. Neutrophils were cultured for 3.5 h at 37°C and 5% CO_2_ (A) without (basal) or with Stx2-producing *E*. *coli* O157:H7 (125/99; STEC), *E*. *coli* O157:H7ΔStx2 devoid of Stx producing capacity (ΔSTEC), or *E*. *coli* C600 (a non-pathogenic strain) at a MOI of 0.5; (B) without (basal) or with STEC, ΔSTEC, ΔSTEC whose supernatant was previously replaced by the medium from a STEC culture (ΔSTEC+STEC sup), or ΔSTEC supplemented with purified Stx2 (0.1 μg/ml; ΔSTEC+Stx2) at MOI 0.5; and the LDH activity (A and B) was determined in culture supernatants. Graphs depict the mean ± SEM of experiments performed in triplicate; each dot represents the triplicate´s mean for each individual donor sample. Statistical significance between samples was assessed by One-way ANOVA followed by Sidak´s multiple comparisons test for data with normal distribution and Friedman test followed by Dunn’s multiple comparisons test for data with non-normal distribution. ns: non-significant.
(JPG)

S2 Fig Compounds employed to inhibit Inflammasome pathway do not induce neutrophil lytic death. Neutrophils were pretreated or not with (**A**) the NLRP3 inhibitor (MCC950, 10 μM); (**B**) the caspase-1 inhibitor Ac-YVAD-CMK (YVAD; 50 μM) or the PAN-caspase inhibitor Z-VAD-FMK (ZVAD, 50μM); or (**C**) the caspase-1/4 inhibitor VX-765 (50 μM), for 30 min and then challenged with STEC (MOI 0.5) for 3.5 h at 37°C and 5% CO_2_. Then, the LDH activity in culture supernatants was determined with an LDH Cytotoxicity Assay Kit. Graphs depict the mean ± SEM of experiments performed in triplicate; each dot represents the triplicate´s mean for each individual donor sample. Statistical significance between samples was assessed by One-way ANOVA followed by Sidak´s multiple comparisons test for data with normal distribution and Friedman test followed by Dunn’s multiple comparisons test for data with non-normal distribution. ns: non-significant.
(TIF)

S3 Fig Neutrophils challenged with ΔSTEC did not have compromised membranes at the end of the coculture. Neutrophils were cultured for 3.5 h at 37°C and 5% CO_2_ without (basal) or with ΔSTEC at a multiplicity of infection (MOI) of 50 for 3.5 h in the presence of Sytox green and live cells images were captured by confocal microscopy. Upper panel shows three representative microscopic fields of unstimulated cells; lower panel shows three representative microscopic fields of neutrophils cultured with the bacteria. Images are representative of experiments performed with 4 donors.
(JPG)

S4 Fig Comparison of the nucleus’ morphology of viable, apoptotic, and necrotic neutrophils with those of neutrophils challenged with STEC. Neutrophils were cultured at 37°C and 5% CO_2_ without (viable neutrophils, basal) or with Stx2-producing *E*. *coli* O157:H7 at MOI 50 (125/99; neutrophils+STEC). Alternatively, neutrophils were left untreated for 18 h to let them undergo apoptosis (apoptotic neutrophils) or were heated at 95°C for 5 min and cultured for 3.5 additional hours to undergo necrosis. At the end of the cultures, neutrophils were fixed with PFA 4%, and stained with propidium iodide. Then, images were captured by confocal microscopy by using a FluoView FV1000 confocal microscope (Olympus, Tokyo, Japan) equipped with a Plapon 60X/1.42 objective. Neutrophil nuclei were pseudo-colored in green.
(TIF)

S5 Fig Comparison of IL-1β secretion at different time points for MOI 0.5 and 50. Neutrophils were cultured for 2 or 3.5 h at 37°C and 5% CO_2_ without (basal) or with STEC at the indicated MOI (0.5 or 50). Then, IL-1β concentrations in culture supernatants were determined by ELISA. Graphs depict the mean ± SEM of experiments performed in triplicate; each dot represents the triplicate´s mean for each individual donor sample. Statistical significance between samples was assessed by Two-way ANOVA followed by Sidak´s multiple comparisons test. *** p<0.001.
(TIF)

S6 Fig Evaluation of the capacity of AEBSF and Ecotin to inhibit human neutrophil elastase activity. Neutrophils were incubated in the presence or absence of AEBSF (0.1 or 1 mM) or Ecotin (100 μg/ml) for 30 minutes at 37°C at 5% CO_2_. Then, cells were lysed with Tritón 0.1% and Ecotin was supplemented to the corresponding treatment after lysis. A dilution (1/5) of lysed cells was incubated with a fluorescent substrate ((Z-Ala-Ala-Ala-Ala)2Rh110) for 1.5 h at 37°C, and then fluorescence at 525 nm was recorded in a Varioskan-Lux plate reader. The graph depicts the mean ± SEM relative fluorescence units (RFU) of independent experiments performed with neutrophils of different donors, each one represented as a dot.
(TIF)

S7 Fig LDH levels in neutrophil supernatants challenged with STEC after different pretreatments. (A) Neutrophils were pretreated or not with Disulfiram (DSF) for 30 min, and then challenged with STEC at the indicated MOI. Three and a half hours later the LDH activity in culture supernatants were determined according to the manufacturer’s instructions and expressed as the % of maximum activity. (B) Neutrophils were stimulated with LPS (150 ng/ml) for 2 h, then were treated with vehicle or ATP and 30 min later were washed and challenged with STEC (MOI 0.5), and 3 h later the LDH activity in culture supernatants was determined. (C) Neutrophils were pretreated or not with DPI (10 μM), VX-765 (50 μM) or both inhibitors together for 30 min, and then challenged with STEC at a MOI of 0.5 for 3 h. Then, the LDH activity in culture supernatants was determined. Graphs depict the mean ± SEM of experiments performed in triplicate; each dot represents the triplicate´s mean for each individual donor sample.
(TIF)

S8 Fig Evaluation of the capacity of STEC to produce Stx when growing in bacterial growth medium (BGM). The Stx2-sensitive Vero cell line was grown in RPMI 1640 medium supplemented with 10% FBS, penicillin, and streptomycin on 96-well plates for 18 h at 37°C. STEC were cultured overnight in BGM (RPMI 1640 without phenol red medium with 0.05% NH_4_Cl and 2% glucose) or with TS (tryptic soy) broth medium at 37°C with shaking at 200 rpm. By the end of the incubation an Optical Density (OD) at 600 nm of 0.9 was reached and the corresponding CFU/ml estimated. Cultures were centrifuged for 10 minutes at 9600 x g and serial dilutions of the supernatants were seeded on the Vero cells monolayers previously grown in 96-well plates and incubated for 48 h at 37°C and 5% CO_2_ for further assessment of Stx presence. Afterward cells were washed with PBS, then fixed and stained for 5 min at room temperature (RT) with 0.1% crystal violet with 20% methanol. Then, wells were washed thrice with fresh water and the remaining cells were solubilized using 30% acetic acid for 20 min at RT. Absorbance at 550 nm was determined in a spectrophotometer as a measurement of cell viability. The graph shows that STEC grown in either BGM or TSB exhibited similar impact on VERO cells viability evaluated as a readout of the presence of Stx2.
(TIF)

S9 Fig STEC, but not ΔSTEC supernatants exerted cytotoxicity on Vero cells. STEC and ΔSTEC were cultured overnight in BGM. Then, supernatants were collected and evaluated for cytotoxic capacity on VERO cell cultures, following the procedures describe in S7 Fig. For easiest illustration, data were depicted as the mortality percentage of VERO cells.
(TIF)
