# Supplementary figures and images for: Regulation of human neutrophil IL-1β secretion induced by Escherichia coli O157:H7 responsible for hemolytic uremic syndrome
Source: PLoS Pathog. 2023 Dec 21;19(12):e1011877. doi: 10.1371/journal.ppat.1011877 (PMC10769087; doi:10.1371/journal.ppat.1011877)

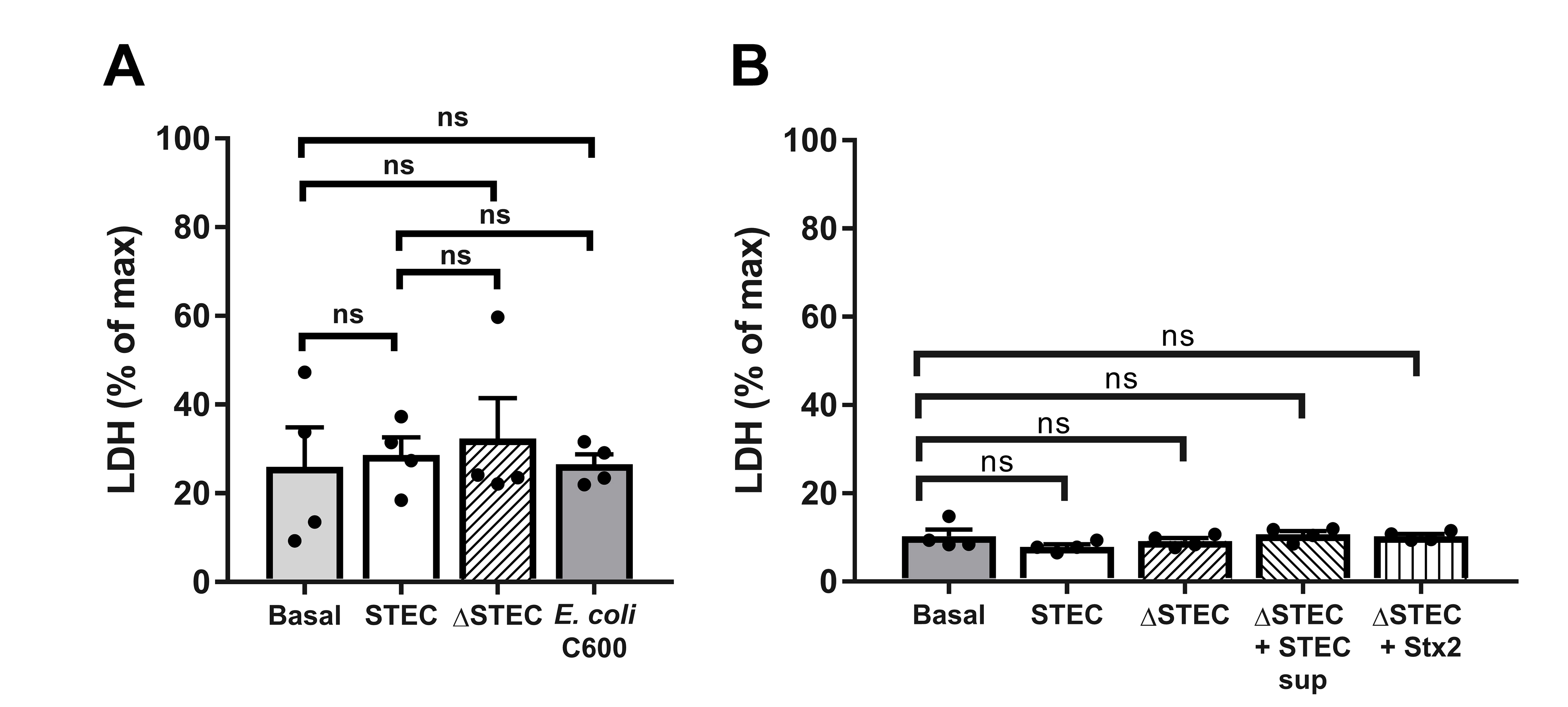

Supplement: S1 Fig — Neutrophils were cultured for 3.5 h at 37°C and 5% CO2 (A) without (basal) or with Stx2-producing E. coli O157:H7 (125/99; STEC), E. coli O157:H7ΔStx2 devoid of Stx producing capacity (ΔSTEC), or E. coli C600 (a non-pathogenic strain) at a MOI of 0.5; (B) without (basal) or with STEC, ΔSTEC, ΔSTEC whose supernatant was previously replaced by the medium from a STEC culture (ΔSTEC+STEC sup), or ΔSTEC supplemented with purified Stx2 (0.1 μg/ml; ΔSTEC+Stx2) at MOI 0.5; and the LDH activity (A and B) was determined in culture supernatants. Graphs depict the mean ± SEM of experiments performed in triplicate; each dot represents the triplicate´s mean for each individual donor sample. Statistical significance between samples was assessed by One-way ANOVA followed by Sidak´s multiple comparisons test for data with normal distribution and Friedman test followed by Dunn’s multiple comparisons test for data with non-normal distribution. ns: non-significant. (JPG) [file ppat.1011877.s002.jpg]

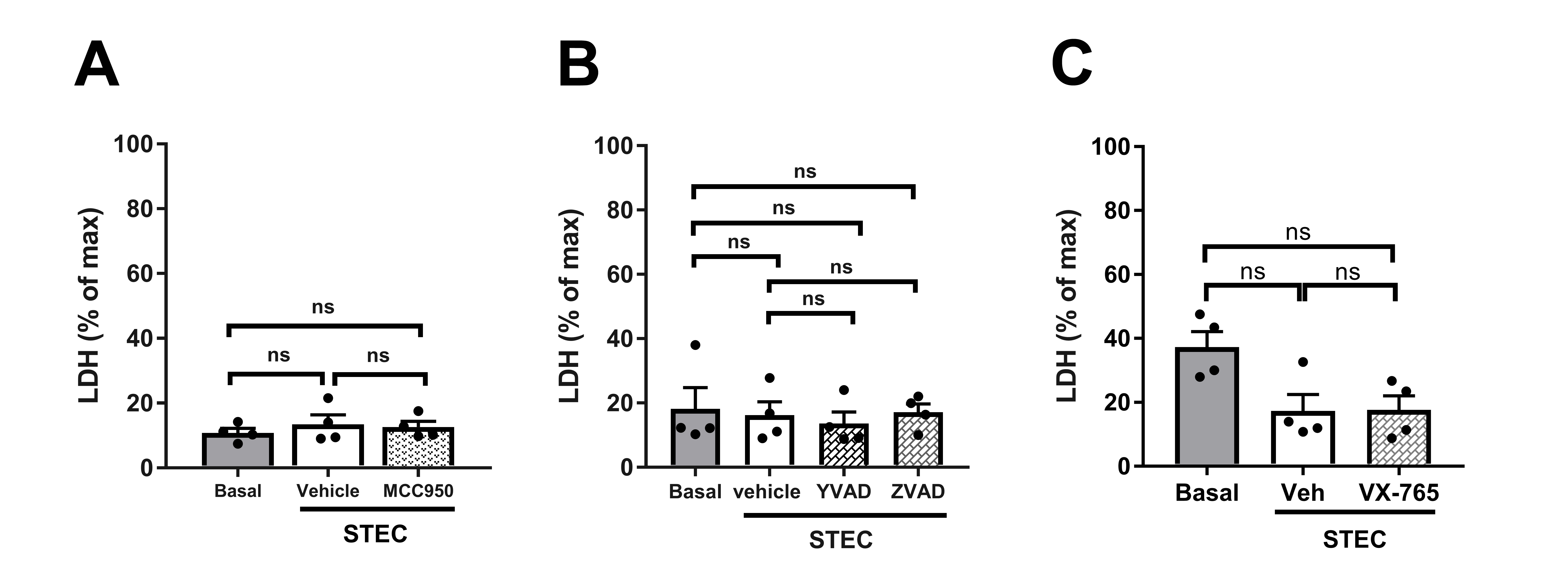

Supplement: S2 Fig — Neutrophils were pretreated or not with (A) the NLRP3 inhibitor (MCC950, 10 μM); (B) the caspase-1 inhibitor Ac-YVAD-CMK (YVAD; 50 μM) or the PAN-caspase inhibitor Z-VAD-FMK (ZVAD, 50μM); or (C) the caspase-1/4 inhibitor VX-765 (50 μM), for 30 min and then challenged with STEC (MOI 0.5) for 3.5 h at 37°C and 5% CO2. Then, the LDH activity in culture supernatants was determined with an LDH Cytotoxicity Assay Kit. Graphs depict the mean ± SEM of experiments performed in triplicate; each dot represents the triplicate´s mean for each individual donor sample. Statistical significance between samples was assessed by One-way ANOVA followed by Sidak´s multiple comparisons test for data with normal distribution and Friedman test followed by Dunn’s multiple comparisons test for data with non-normal distribution. ns: non-significant. (TIF) [file ppat.1011877.s003.tif]

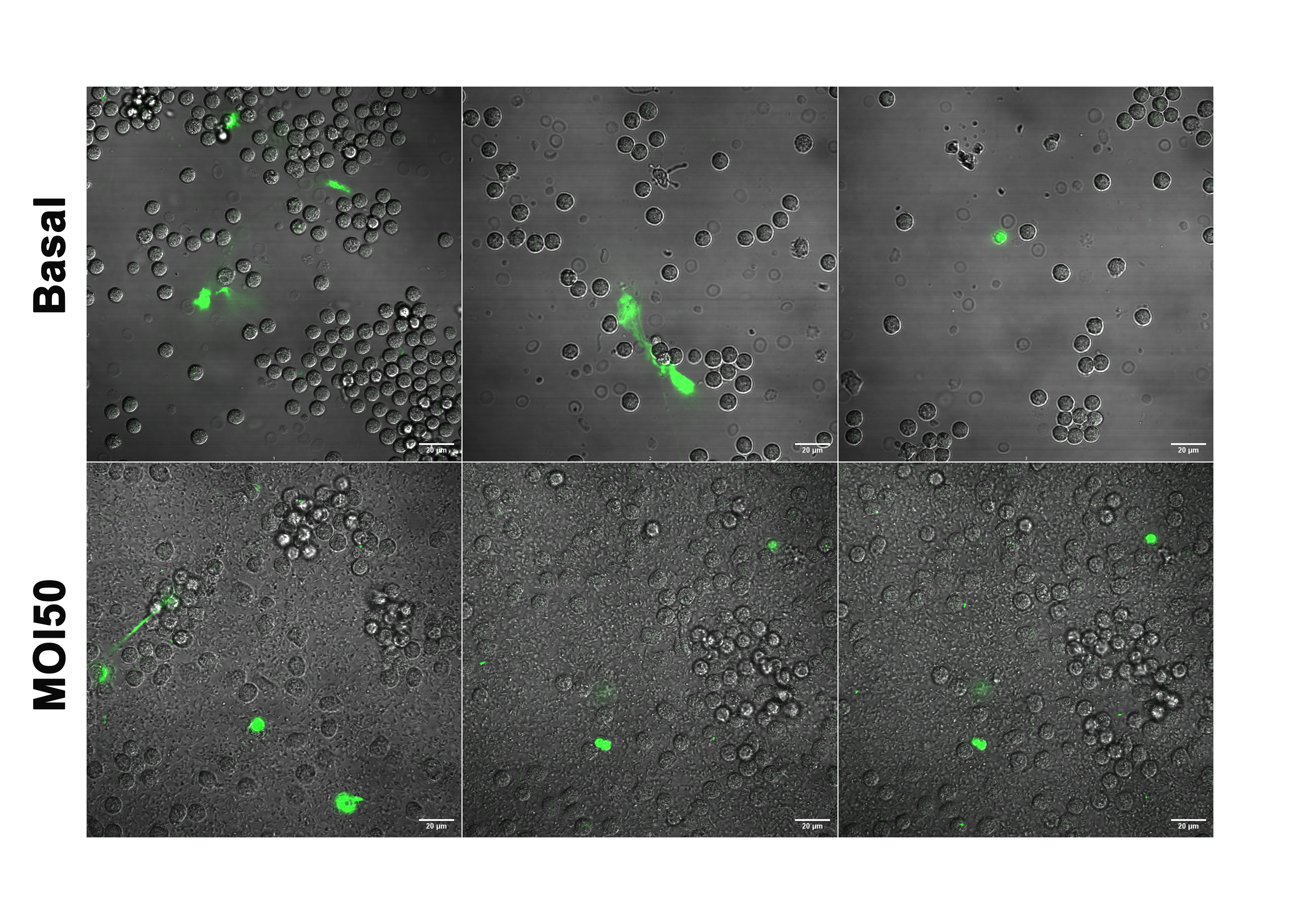

Supplement: S3 Fig — Neutrophils were cultured for 3.5 h at 37°C and 5% CO2 without (basal) or with ΔSTEC at a multiplicity of infection (MOI) of 50 for 3.5 h in the presence of Sytox green and live cells images were captured by confocal microscopy. Upper panel shows three representative microscopic fields of unstimulated cells; lower panel shows three representative microscopic fields of neutrophils cultured with the bacteria. Images are representative of experiments performed with 4 donors. (JPG) [file ppat.1011877.s004.jpg]

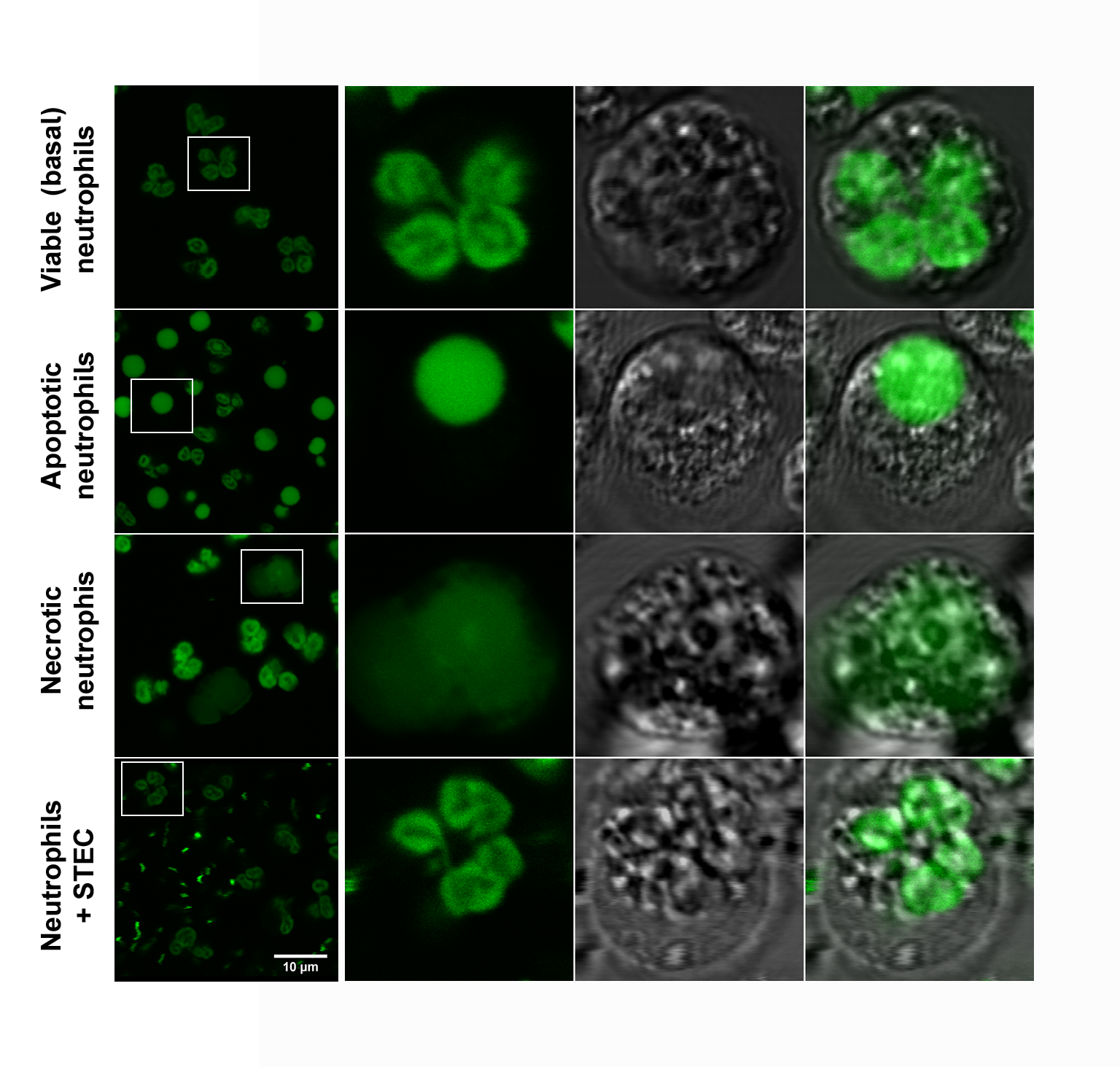

Supplement: S4 Fig — Neutrophils were cultured at 37°C and 5% CO2 without (viable neutrophils, basal) or with Stx2-producing E. coli O157:H7 at MOI 50 (125/99; neutrophils+STEC). Alternatively, neutrophils were left untreated for 18 h to let them undergo apoptosis (apoptotic neutrophils) or were heated at 95°C for 5 min and cultured for 3.5 additional hours to undergo necrosis. At the end of the cultures, neutrophils were fixed with PFA 4%, and stained with propidium iodide. Then, images were captured by confocal microscopy by using a FluoView FV1000 confocal microscope (Olympus, Tokyo, Japan) equipped with a Plapon 60X/1.42 objective. Neutrophil nuclei were pseudo-colored in green. (TIF) [file ppat.1011877.s005.tif]

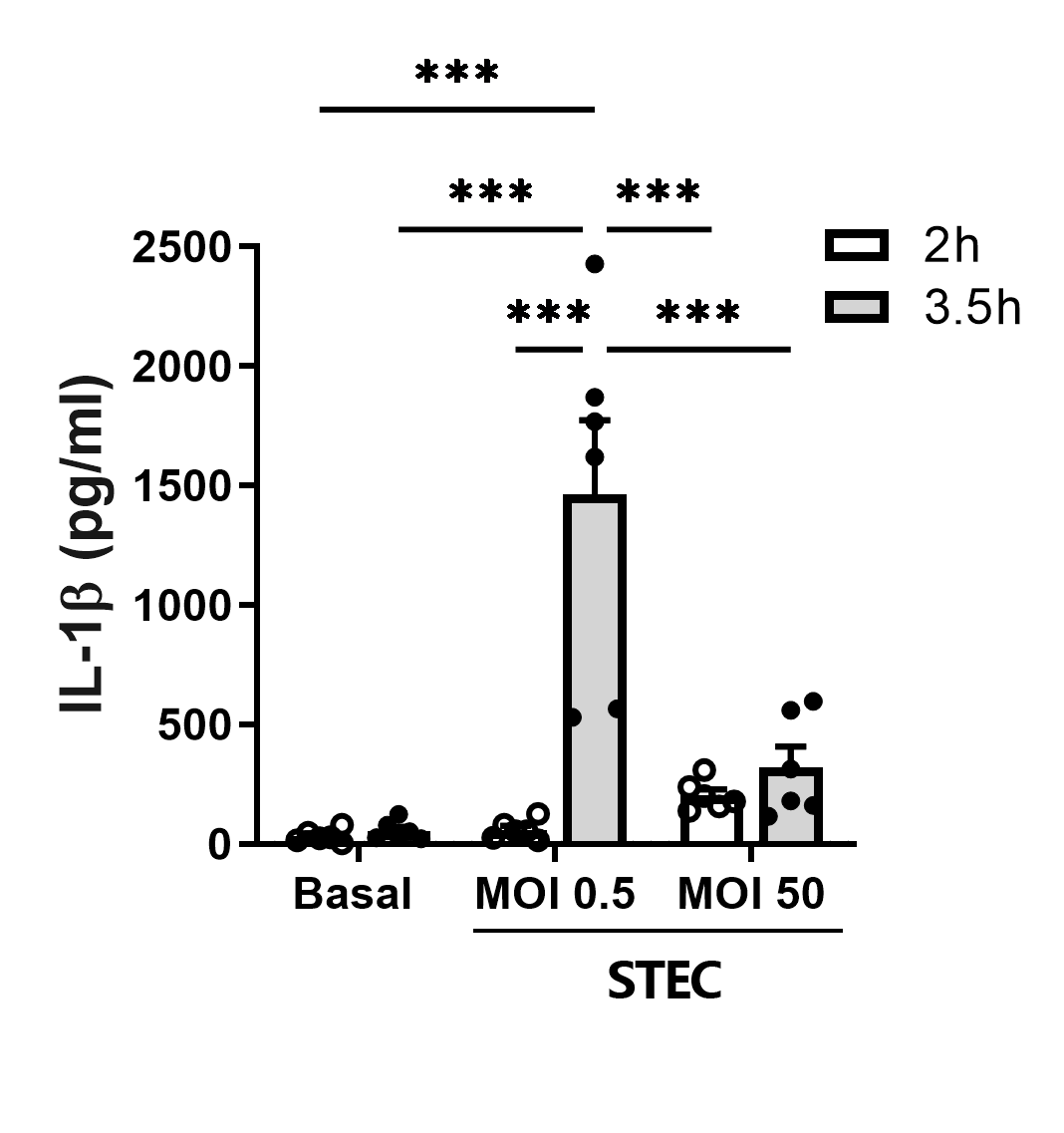

Supplement: S5 Fig — Neutrophils were cultured for 2 or 3.5 h at 37°C and 5% CO2 without (basal) or with STEC at the indicated MOI (0.5 or 50). Then, IL-1β concentrations in culture supernatants were determined by ELISA. Graphs depict the mean ± SEM of experiments performed in triplicate; each dot represents the triplicate´s mean for each individual donor sample. Statistical significance between samples was assessed by Two-way ANOVA followed by Sidak´s multiple comparisons test. *** p<0.001. (TIF) [file ppat.1011877.s006.tif]

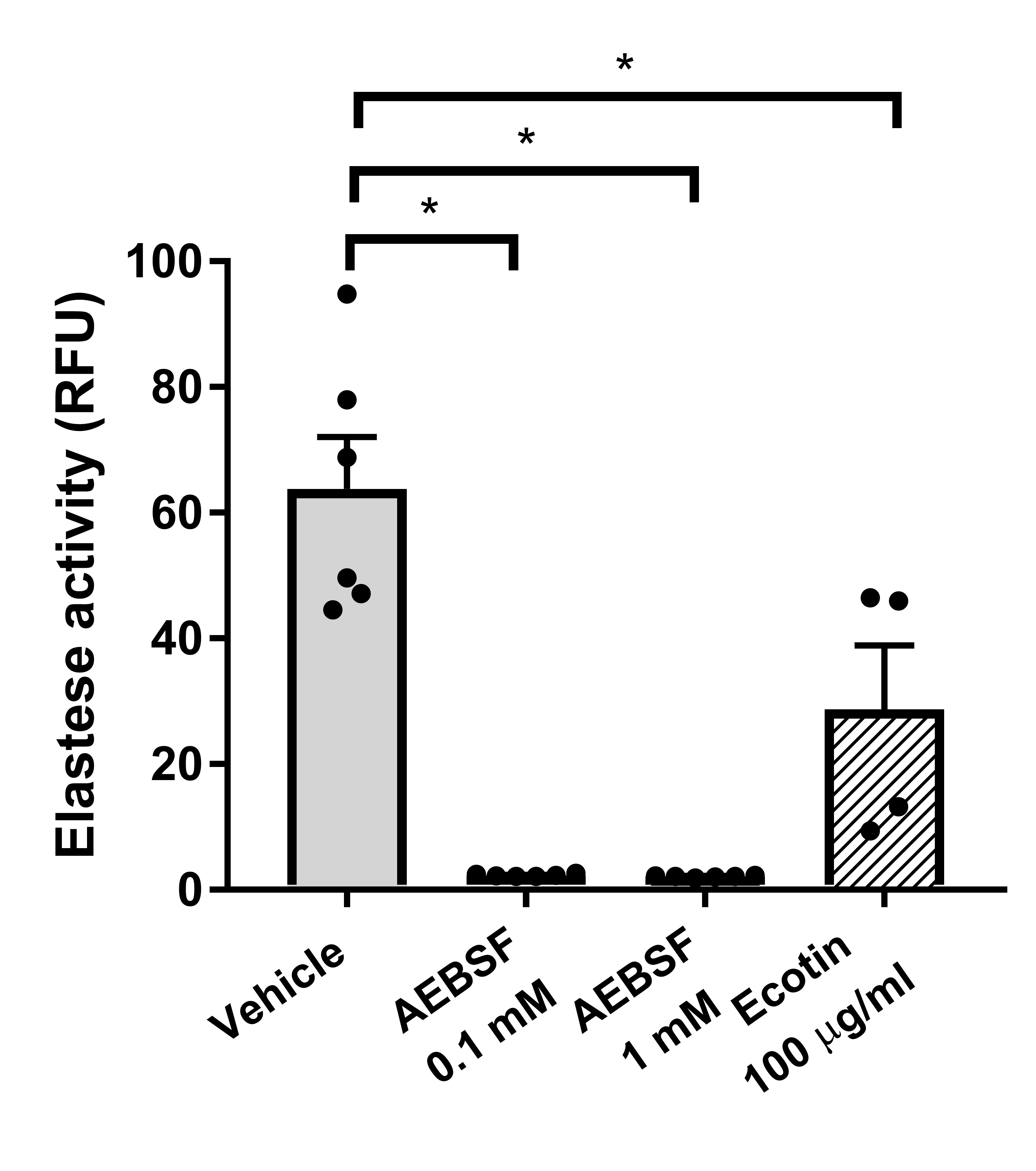

Supplement: S6 Fig — Neutrophils were incubated in the presence or absence of AEBSF (0.1 or 1 mM) or Ecotin (100 μg/ml) for 30 minutes at 37°C at 5% CO2. Then, cells were lysed with Tritón 0.1% and Ecotin was supplemented to the corresponding treatment after lysis. A dilution (1/5) of lysed cells was incubated with a fluorescent substrate ((Z-Ala-Ala-Ala-Ala)2Rh110) for 1.5 h at 37°C, and then fluorescence at 525 nm was recorded in a Varioskan-Lux plate reader. The graph depicts the mean ± SEM relative fluorescence units (RFU) of independent experiments performed with neutrophils of different donors, each one represented as a dot. (TIF) [file ppat.1011877.s007.tif]

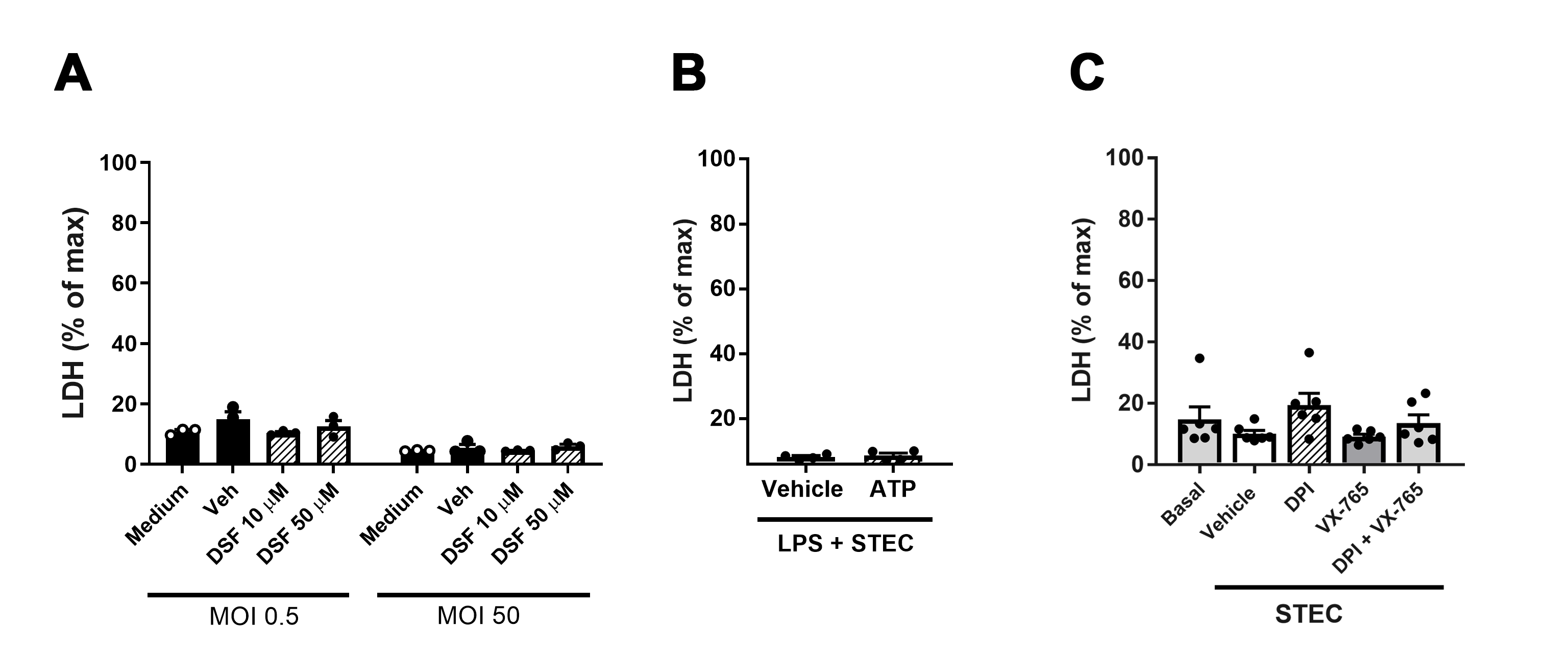

Supplement: S7 Fig — (A) Neutrophils were pretreated or not with Disulfiram (DSF) for 30 min, and then challenged with STEC at the indicated MOI. Three and a half hours later the LDH activity in culture supernatants were determined according to the manufacturer’s instructions and expressed as the % of maximum activity. (B) Neutrophils were stimulated with LPS (150 ng/ml) for 2 h, then were treated with vehicle or ATP and 30 min later were washed and challenged with STEC (MOI 0.5), and 3 h later the LDH activity in culture supernatants was determined. (C) Neutrophils were pretreated or not with DPI (10 μM), VX-765 (50 μM) or both inhibitors together for 30 min, and then challenged with STEC at a MOI of 0.5 for 3 h. Then, the LDH activity in culture supernatants was determined. Graphs depict the mean ± SEM of experiments performed in triplicate; each dot represents the triplicate´s mean for each individual donor sample. (TIF) [file ppat.1011877.s008.tif]

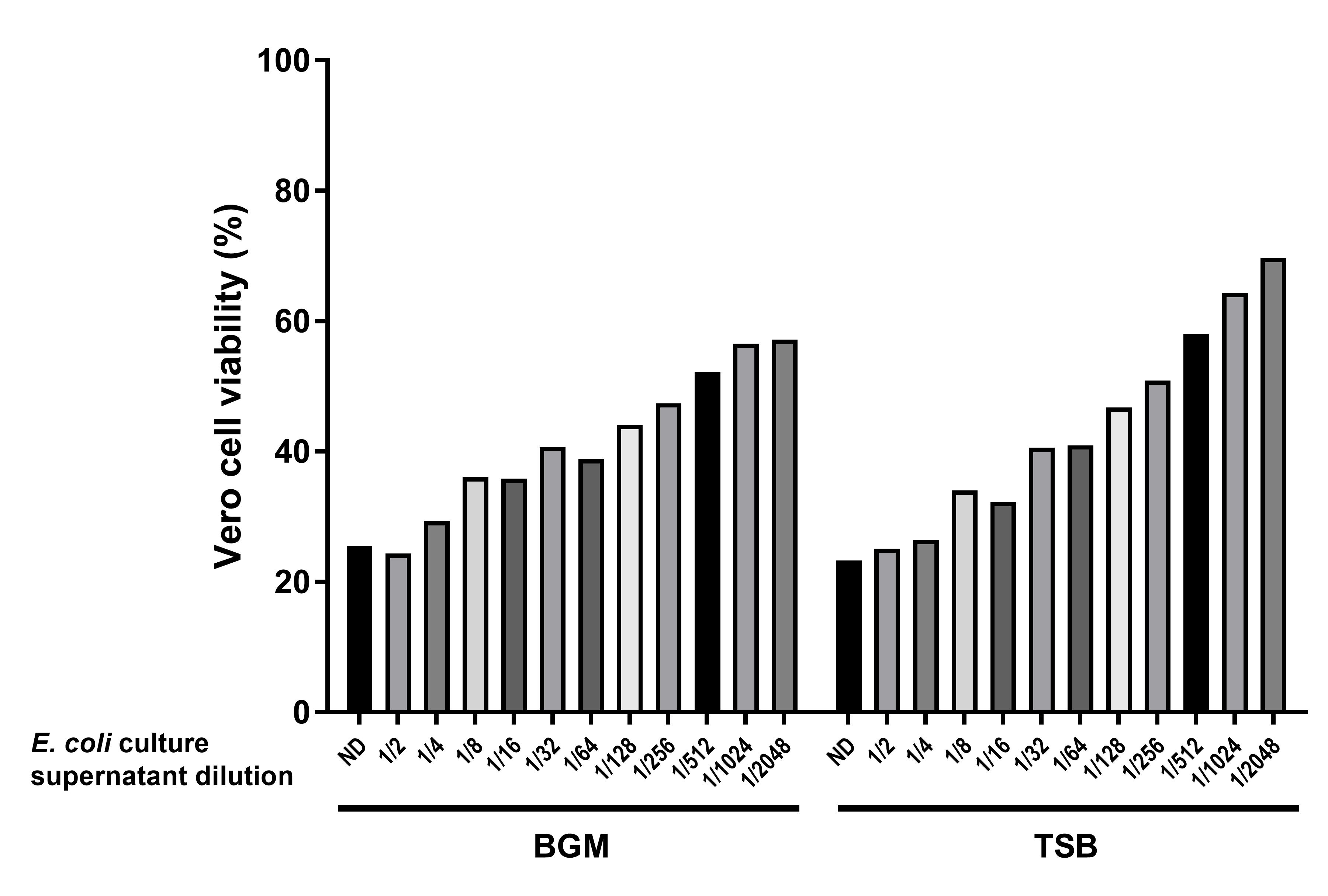

Supplement: S8 Fig — The Stx2-sensitive Vero cell line was grown in RPMI 1640 medium supplemented with 10% FBS, penicillin, and streptomycin on 96-well plates for 18 h at 37°C. STEC were cultured overnight in BGM (RPMI 1640 without phenol red medium with 0.05% NH4Cl and 2% glucose) or with TS (tryptic soy) broth medium at 37°C with shaking at 200 rpm. By the end of the incubation an Optical Density (OD) at 600 nm of 0.9 was reached and the corresponding CFU/ml estimated. Cultures were centrifuged for 10 minutes at 9600 x g and serial dilutions of the supernatants were seeded on the Vero cells monolayers previously grown in 96-well plates and incubated for 48 h at 37°C and 5% CO2 for further assessment of Stx presence. Afterward cells were washed with PBS, then fixed and stained for 5 min at room temperature (RT) with 0.1% crystal violet with 20% methanol. Then, wells were washed thrice with fresh water and the remaining cells were solubilized using 30% acetic acid for 20 min at RT. Absorbance at 550 nm was determined in a spectrophotometer as a measurement of cell viability. The graph shows that STEC grown in either BGM or TSB exhibited similar impact on VERO cells viability evaluated as a readout of the presence of Stx2. (TIF) [file ppat.1011877.s009.tif]

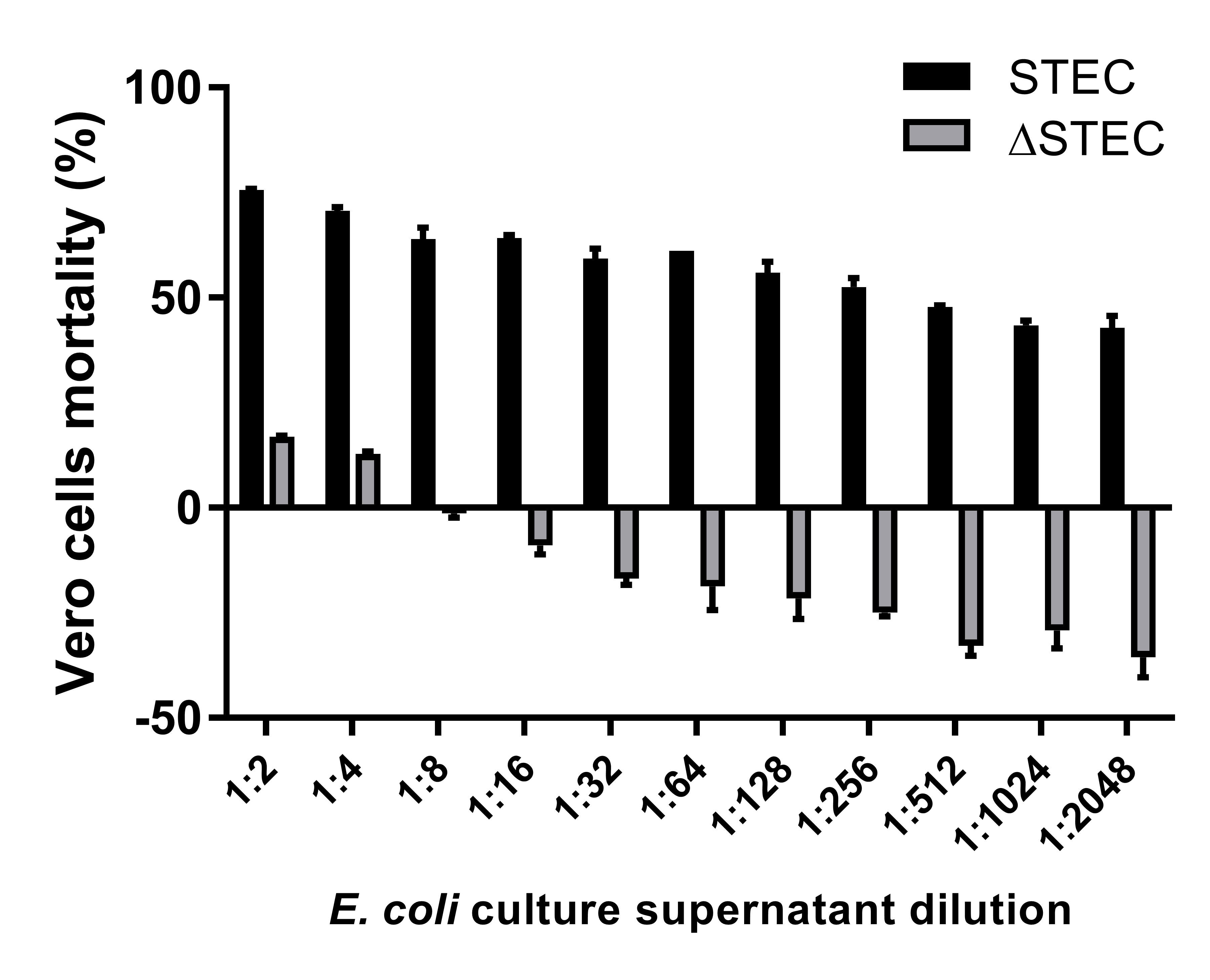

Supplement: S9 Fig — STEC and ΔSTEC were cultured overnight in BGM. Then, supernatants were collected and evaluated for cytotoxic capacity on VERO cell cultures, following the procedures describe in S7 Fig. For easiest illustration, data were depicted as the mortality percentage of VERO cells. (TIF) [file ppat.1011877.s010.tif]
